# Varying benefits of generalist and specialist camouflage in two versus four background environments

**DOI:** 10.1093/beheco/arac114

**Published:** 2023-03-26

**Authors:** Anna E Hughes, Emmanuelle S Briolat, Lina María Arenas, Eric Liggins, Martin Stevens

**Affiliations:** Centre for Ecology and Conservation, University of Exeter, Penryn Campus, Penryn TR10 9FE, UK; Department of Psychology, University of Essex, Wivenhoe House, Colchester CO4 3SQ, UK; Centre for Ecology and Conservation, University of Exeter, Penryn Campus, Penryn TR10 9FE, UK; Centre for Ecology and Conservation, University of Exeter, Penryn Campus, Penryn TR10 9FE, UK; QinetiQ, Cody Technology Park, Ively Road, Farnborough, Hampshire GU14 0LX, UK; Centre for Ecology and Conservation, University of Exeter, Penryn Campus, Penryn TR10 9FE, UK

**Keywords:** background matching, camouflage, crypsis, generalist, specialist

## Abstract

Background-matching camouflage is a well-established strategy to reduce detection, but implementing this on heterogeneous backgrounds is challenging. For prey with fixed color patterns, solutions include specializing on a particular visual microhabitat, or adopting a compromise or generalist appearance, matching multiple backgrounds less well. Existing studies suggest both approaches can succeed, but most consider relatively simple scenarios, where artificial prey appear against two backgrounds differing in a single visual characteristic. Here, we used computer-based search tasks with human participants to test the relative benefits of specializing and generalizing for complex targets, displayed on either two or four types of naturalistic backgrounds. Across two background types, specialization was beneficial on average. However, the success of this strategy varied with search duration, such that generalist targets could outperform specialists over short search durations due to the presence of poorly matched specialists. Over longer searches, the remaining well-matched specialists had greater success than generalists, leading to an overall benefit of specialization at longer search durations. Against four different backgrounds, the initial cost to specialization was greater, so specialists and generalists ultimately experienced similar survival. Generalists performed better when their patterning was a compromise between backgrounds that were more similar to each other than when backgrounds were more different, with similarity in luminance more relevant than pattern differences. Time dependence in the relative success of these strategies suggests that predator search behavior may affect optimal camouflage in real-world situations.

## Teaser

When hiding in a variable environment, it is usually best to specialize and match part of the background scene. Although this depends on properties of the background, and for how long predators will search. In games where human “predators” looked for artificial targets, we found that specialists had an advantage over generalists (who matched several different backgrounds). However, generalists did better across relatively similar backgrounds, or across more than two backgrounds, and they outperformed specialists in the first seconds of searching.

## INTRODUCTION

The mechanisms underlying the effectiveness of visual camouflage are a focus of much recent study (Cuthill 2019), both with regard to understanding the evolution of anti-predator defences in animals, and its relevance to human contexts, such as the military. Perhaps the most fundamental camouflage strategy is background matching: where an animal or other object matches the general color, luminance, and pattern of the background (Endler 1978, 1984; Stevens and Merilaita 2011; Nokelainen and Stevens 2016; Merilaita et al. 2017). Background matching has been demonstrated to be effective in a number of species, reducing detectability by potential predators and improving survival (Kang et al. 2012; Lovell et al. 2013; Marshall et al. 2016; Troscianko et al. 2016; Duarte et al. 2018; Orton et al. 2018; Walton and Stevens 2018). However, background matching has important limitations: in particular, animals commonly move between habitats, or live in environments that are naturally heterogeneous, making it challenging to accurately match the background at all times. In some cases, animals may be able to change their color (Duarte et al. 2017), body patterning (Hanlon 2007) or adjust their behavior (Stevens and Ruxton 2018), in order to achieve a better match. However, many animals have fixed color patterns and behavioral choices are unlikely to always fully mitigate the risks of poor background matching. As such, how camouflage should be optimized against variable and heterogeneous backgrounds remains an important question (Hughes et al. 2019).

A number of studies have addressed the above question theoretically (Merilaita et al. 1999; Houston et al. 2007). For an animal needing to move between two different habitats, one possible strategy is to specialize, resembling one of the backgrounds as closely as possible but potentially at the expense of not closely matching the other background. Despite the likely cost of higher visibility on the mismatched background, this strategy could be advantageous if the benefit of increased survival on the matching background is higher than this cost. Alternatively, an animal could generalize, evolving “imperfect camouflage” that partially matches both backgrounds. Theoretical modeling predicts that the best strategy will vary according to a number of extraneous factors, linked to the environment’s structure and the behavior of relevant predators, as well as properties of the prey animals themselves (Merilaita et al. 1999; Houston et al. 2007). However, all else being equal, the optimal strategy should be determined by the trade-off between crypsis in one microhabitat versus the other, which in turn depends on background similarity (Merilaita et al. 1999). When the two backgrounds are similar, improving the match to one may only slightly reduce crypsis in the other, facilitating a generalist strategy. By contrast, for more dissimilar backgrounds, any effective match to one may substantially increase conspicuousness against the other, such that successful camouflage on both backgrounds is limited, and a specialist strategy is predicted to be most effective.

New methods for quantifying camouflage and the similarity between prey animals and their natural habitats, from the perspective of potential predators, have shown that many species are locally adapted to specific backgrounds (Harris and Weatherall 1991; Boratyński et al. 2014; Marshall et al. 2015; Niu et al. 2017; Yamamoto and Sota 2020), and that these small improvements in background matching can have beneficial effects on survival (Troscianko et al. 2016). Yet, despite the difficulty of conclusively demonstrating that natural color patterns operate as generalists (Cuthill 2019), putative generalist morphs are thought to exist in several species, including shrimp (Duarte et al. 2016), crabs (Nokelainen et al. 2019), and skinks (Baling et al. 2020), and there is evidence that imperfect camouflage can provide some protection from observers (Nokelainen et al. 2019, 2020; Rodríguez-Gironés and Maldonado 2020; Barnett et al. 2021). In turn, empirical tests using artificial setups of the effectiveness of generalist and specialist strategies in nonhuman animals broadly support the predictions of theoretical models, suggesting that compromise patterns can be successful, and that their relative benefits depend on background similarity (Merilaita et al. 2001; Bond and Kamil 2006). Similar results have been found in experiments with human volunteers acting as “predators” in computer and web-based search tasks. In one study, where targets evolved in response to the reaction times of participants playing a web-based game, generalist and specialist forms performed similarly when the backgrounds were distinguished by the size of the background elements (Sherratt et al. 2007). In another experiment, generalists (with intermediate elements) survived better than specialist targets (with elements of the same size as one of the backgrounds) when the size difference between the backgrounds was relatively small (Toh and Todd 2017). However, as the pattern size difference between the backgrounds increased, the two strategies became equivalent, indicating that generalist strategies offer better protection against predation when the backgrounds are relatively similar.

While these studies have helped to verify the main theoretical predictions, a number of key questions remain. First, many of the experiments were conducted using relatively small numbers of target and background types, often with fairly simple, artificial patterning that varied on only one dimension, usually pattern size (but see Karpestam et al. 2013). By contrast, naturalistic backgrounds vary in complex ways in color, luminance, and spatial frequency. It is therefore important to ask whether background similarity is able to predict the optimal strategy with more realistic visual stimuli. So far, when studies testing target detection in screen-based tasks have used natural backgrounds, these have been limited in scope, mostly based on images of tree bark (Troscianko et al. 2013, 2018; Michalis et al. 2017; Troscianko, Skelhorn, et al. 2017). Second, it is of interest to consider exactly what aspects of the background might be most important in determining whether backgrounds can be considered “similar.” For example, in web-based search tasks where generalist and specialists performed similarly when the backgrounds differed in pattern, contrasting results were found when the backgrounds varied in luminance, with specialists gaining an advantage, suggesting that different aspects of the backgrounds can select for different outcomes (Sherratt et al. 2007). If receivers are more sensitive to color differences than to size differences, specialist strategies may offer better protection even when the color differences between backgrounds are fairly modest, while generalist strategies may continue to be effective even with larger differences in pattern size (Hughes et al. 2019). Finally, work to date has been limited to the simplest scenario, where targets can be found on two different backgrounds. However, it is possible that generalist strategies may become a better option if targets are regularly encountered on a wider range of backgrounds (Ruxton et al. 2004; Hughes et al. 2019), and many natural habitats will comprise a wide spectrum of possible background types.

In this present study, we address these outstanding questions by developing online “citizen science” computer experiments, similar in general design to earlier search tasks testing principles of camouflage (Troscianko et al. 2013; Troscianko, Skelhorn, et al. 2017) and online experiments investigating detectability of natural stimuli (Troscianko, Wilson-Aggarwal, et al. 2017; Nokelainen et al. 2019; Niu et al. 2021), where human participants search for hidden moth-like triangular targets on a wide range of naturalistic background images. In the first experiment, we presented targets, varying in multiple aspects of color and pattern, against pairs of background types chosen from a potential eight different background categories, and tested whether specialist or generalist strategies were more effective overall. The background combinations used were not designed to mimic any particular natural habitat scenarios, and instead aimed to test general principles, though many of the combinations were plausible (e.g., grassy sand dunes, rock pools). Some man-made backgrounds were also included, as these can form part of the natural environment for animals (e.g., pavements for crickets; Karpestam et al. 2013). We also asked whether background differences could predict the optimal strategy, in line with expectations from theoretical work (Merilaita et al. 1999; Houston et al. 2007), then explored which aspects of visual difference were most important for explaining our results. We predicted that specialist strategies would be most effective when the backgrounds were more different, and generalist strategies more effective when the backgrounds were relatively similar. In the second experiment, we displayed targets against four different background types, to test whether this changed the relative efficacy of specialist and generalist targets. We predicted that generalists might have a bigger advantage in this experiment, given that specialist targets would be well matched on a smaller proportion of backgrounds.

## METHODS

### Experiment 1 (two-background)

In Experiment 1, our two main questions were 1) is a specialist or generalist strategy more successful overall? and 2) how do background properties affect the specialist generalist trade-off? Targets were made from blends of two backgrounds and were designed to either be specialists (better matching one of the two backgrounds used for creation) or generalists (a 50–50 blend between both backgrounds). To address question 1, we asked whether the survival advantage a specialist target obtains on its matching background outweighs the cost on the nonmatching background, in comparison to the generalist target. To address question 2, we asked whether differential survival probability can be predicted by the visual difference between backgrounds.

### Background and target creation

We developed computer experiments in which participants searched for moth-shaped targets hidden in photographs of real natural substrates. Photographs were taken from a bank of images covering a range of natural and man-made background types: leaves, bark, grass, sand, shrubs, pebbles, stone, and brick (see Supplementary Material, Sample backgrounds for examples and further details of photography). Thirty-two images were used for each background type, giving a total of 256 original images.

**Figure 1:**
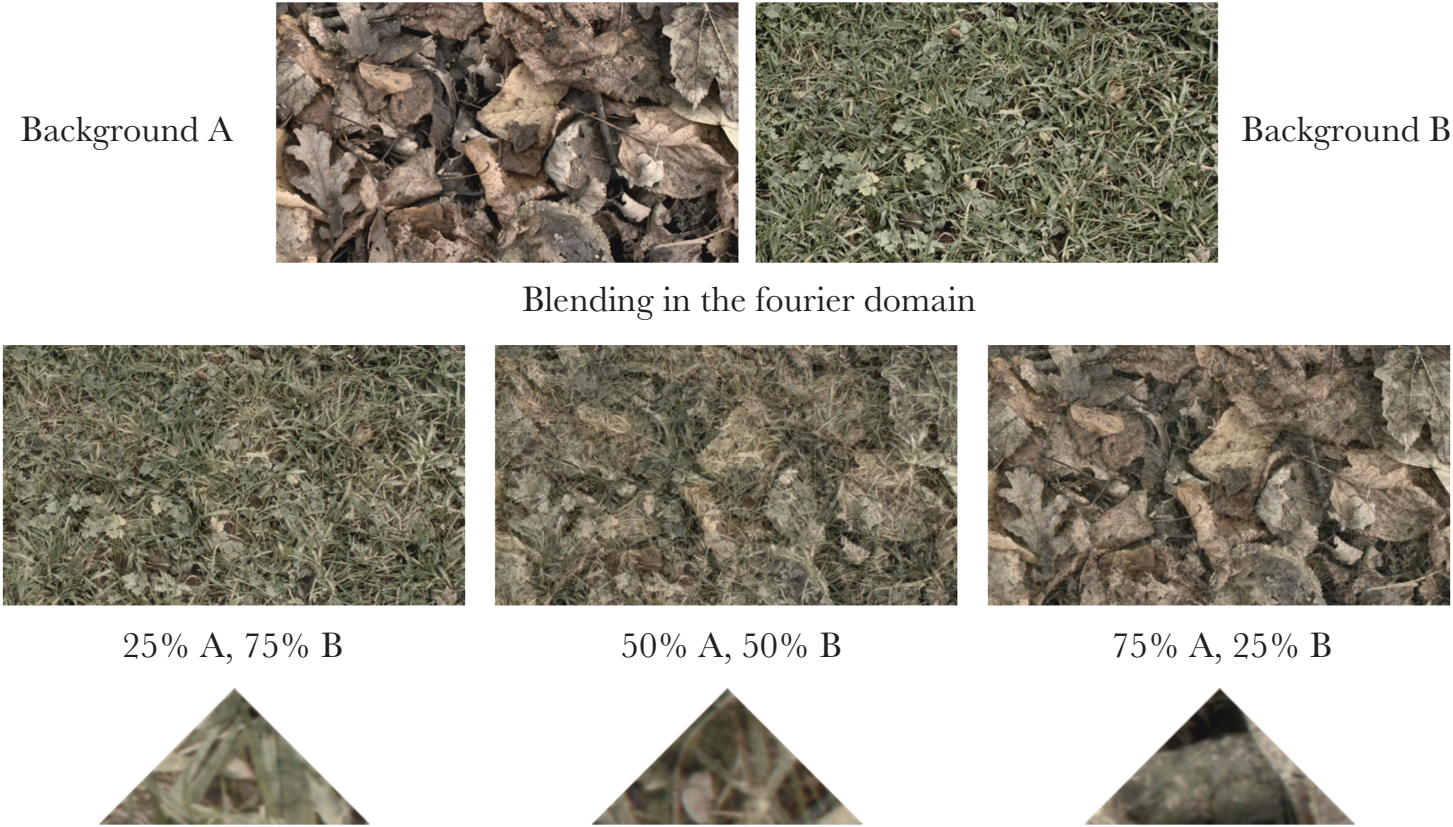
Schematic to show how the targets were created from the backgrounds. Two random backgrounds were selected (a leaf background A and a grass background B in this example, top row) and then blended in the Fourier domain in three different ways: 25% background A and 75% background B (left middle), 50% background A and 50% background B (center middle), and 75% background A and 25% background B (right middle). A randomly placed triangular “moth” target was then cut out from each blended background (bottom row).

Images were read into a custom MATLAB program, allowing us to blend different images together (Stevens and Cuthill 2006; Hughes et al. 2019). The program read in two randomly chosen images, both images were then split into their three color channels (“red,” “green,” and “blue” defined by the camera sensor), and each channel was separately Fast Fourier Transformed. The images were then combined using different weights (using both phase and amplitude components) to give three different “blended” backgrounds: the first contained 25% of background A and 75% of background B (specialist on B), the second contained 50% of backgrounds A and B (generalist), and the third contained 75% of background A and 25% of background B (specialist on A). Once the images were blended, a new image was created by taking the inverse Fourier transform and recombining the three image channels. This blending in the frequency (Fourier transformed) domain ensures that spatial characteristics are retained, even if the starting images are out of phase, while ensuring that the spatial frequencies present in the targets reflect the weightings that is the specialist on B would have more similarity with background B. The process was repeated with a new pair of images to create each set of stimuli, meaning that no two stimuli in the experiments were the same. In total, 766 different image pairs were used, producing 2298 unique blended backgrounds. A “moth” target was then generated from each blended background. The targets therefore resembled a “blend” of both backgrounds, but in the case of the specialist targets, the features of the dominant background would be stronger (see Figure 1 for example stimuli). This was done in ImageJ (Schneider et al. 2012) by randomizing selection of a triangular target 150 pixels wide and 75 pixels high from each image.

### Experiment design

**Figure 2:**
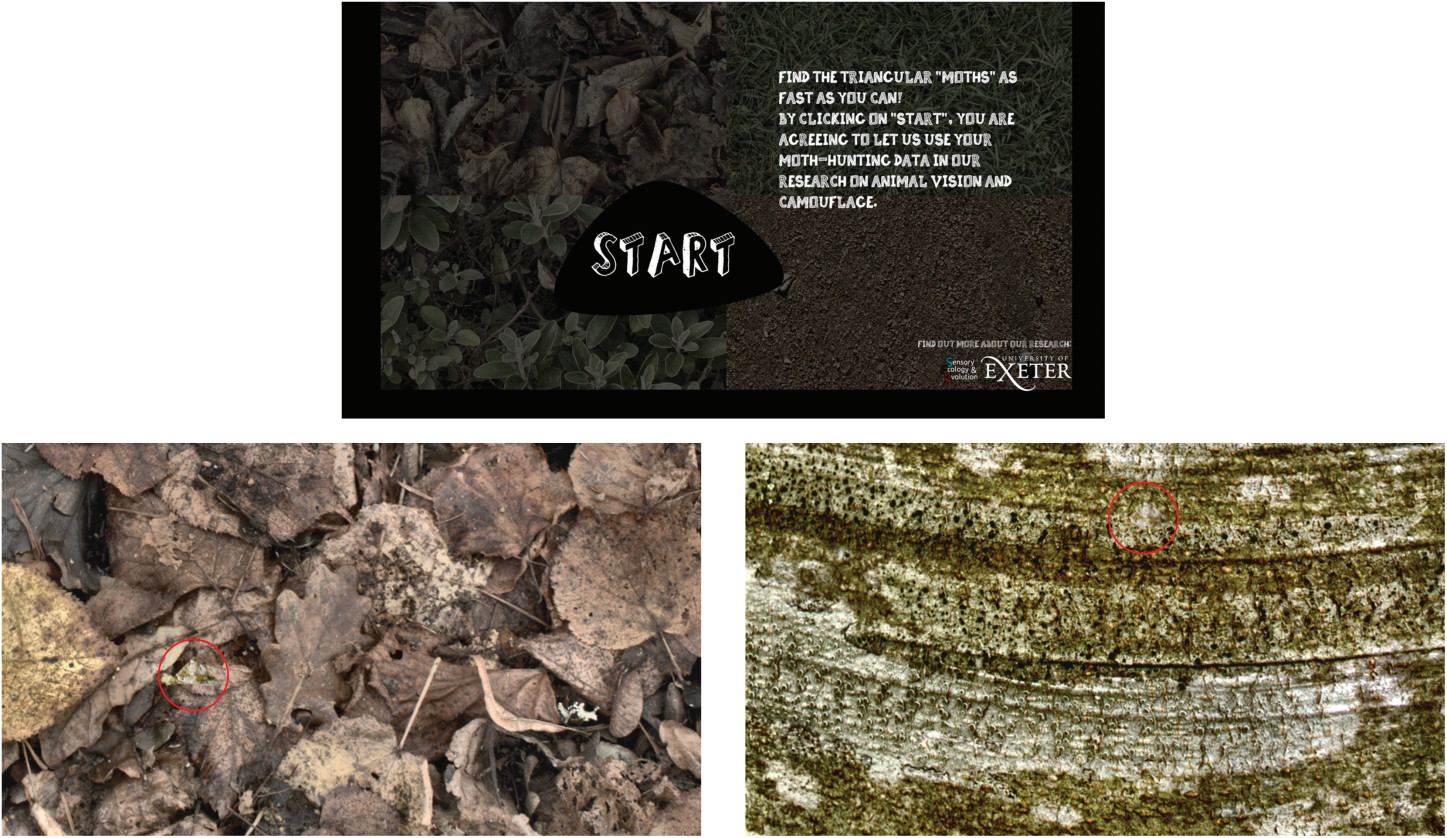
Top: loading screen for the online experiment. Bottom: example trials, showing the same target on the two different backgrounds used to create it (left a leaf background, right a bark background).

The online experiment was freely playable on internet browsers and was created using custom JavaScript code (available at http://camo.sensoryecology.com; Figure 2). Participants viewed 12 slides in total. Six targets were randomly selected for each participant (two from each target condition, i.e., generalist and specialist), and these targets were then viewed on both of the original backgrounds that were used to make them. The order of the slides was randomized so that it was unlikely that participants viewed the same individual targets sequentially. The position of each target on the slide was pseudo-randomized, with the proviso that the two presentations of each target moth were constrained to be at the same radius from the center of the screen. This was undertaken to control for any effect of distance from the screen center, previously shown to be a strong predictor of response time in similar experiments (Troscianko, Wilson-Aggarwal, et al. 2017), as eye movements are biased to the center of the screen in experimental tasks (Bindemann 2010). We also weighted the probability of each possible distance from the center of the screen, making it more likely that targets were found further away from the center and less likely that they were found close to the center, to ensure that the task was sufficiently difficult (as centrally placed targets are much easier to find).

Participants were instructed to hunt for the triangular “moths” and had 15 s on each slide to find the target. They received feedback on each trial: if they located the target, a “positive” sound was played, a green circle appeared around the target and the background faded away to reveal the target. If they clicked in the wrong location, a “negative” sound was played. If they did not locate it in time, the circle was red instead of green. The position of all clicks and their times (including misses) were recorded.

We also carried out a classroom-based version of this experiment with additional conditions, where participants played in a more controlled setting. Methods and results can be found in the Supplementary Material (Experiment 1B) and show a similar pattern of results.

### Experiment 2 (four-background)

To extend the relevance of Experiment 1 to scenarios with multiple different backgrounds, we designed a similar online experiment, in which specialist and generalist targets were presented on four, instead of two, types of natural backgrounds. This aimed to test whether a generalist strategy would be more successful when seen against a wider range of backgrounds.

### Background and target creation

Images were taken from the same bank of photographs used in Experiment 1, processed as above, but were restricted to 30 samples each from four background types (leaves, bark, grass, and shrubs, *N*_TotalImages_ = 120), representing realistic natural backgrounds on which moth-like prey might be found. The same custom MATLAB and ImageJ routines were used to generate three different types of targets from these images: specialists (S), generalists across two backgrounds (G2), and generalists across four backgrounds (G4). Specialist targets were made by selecting three randomly positioned triangular targets per background image, generating 90 targets each per natural background type (*N*_S_ = 360). For G2 targets, each image of a given background type was randomly paired with two images from every other background type to create 50% blends of every possible combination, from which a single randomly positioned triangular target was extracted, producing 60 independent targets for each combination (*N*_G1_ = 360). Finally, pairwise 50% blends of bark and grass images and of leaves and shrub images were again blended together equally to produce G4 images, composed of all four background types; each individual background image was used 12 times, in combination with randomly selected images from the other background types, creating 360 unique blended images, from each of which a single randomly positioned triangular target was taken (*N*_G2_ = 360) (see the Supplementary Material, Experiment 2 for a figure containing example targets and a schematic of how they were created).

### Experiment design

The online experiment was created by adapting the JavaScript code for Experiment 1, and again made freely playable on internet browsers (available at http://camostrategy.sensoryecology.com). Overall experiment design closely followed that of Experiment 1, with some minor modifications to accommodate multiple background types. Each participant was randomly assigned one of 120 sets of targets, containing three randomly selected targets of every type (S, G2, and G4). Each of these nine moths was displayed on a randomly selected image from all four background types, so every participant viewed 36 slides in total. Slide order was randomized, as was the position of the target. Following Experiment 1, participants had 15 s to locate each target, the position and timing of all clicks were recorded, and feedback on success was provided with the same timer, sounds, and circles highlighting the target.

We also carried out a lab-based equivalent to this experiment, using exactly the same background and target images, in which participants all used the same computer setup, to validate the approach in a more controlled setting; methods and results, showing similar trends, are reported in the Supplementary Material (Experiment 2B).

### Participants

We describe each completed experiment as a “play,” and each slide shown (with a background image and target) as a “trial” in the search task. There were 3193 total plays in Experiment 1 (with 1783 unique players) and 1228 plays in Experiment 2 (with 940 unique players). These studies were approved by [University of Exeter]’s Biosciences ethics committee (2018/2332), following principles in the declaration of Helsinki. All participants in Experiments 1 and 2 were recruited online via social media/word-of-mouth and consented to take part in these experiments by clicking the “start” button. They were free to leave the study without specifying a reason at any time; data were only recorded upon pressing the “finish” button at the end of the experiments. Participants were asked to indicate whether they had played before, allowing us to identify unique players. We analyzed all data (regardless of whether the participant indicated they had played before) as the design of the experiment meant that even with repeated playing, participants were unlikely to see the same targets. However, removing repeat plays from the analysis did not change the main conclusions (see Supplementary Material—Additional analyses 1).

Participants playing on mobile devices were excluded due to the small screen size. In Experiment 1, this reduced the sample size to 3106 total plays, with 1717 unique players; in Experiment 2, there were 1204 plays with 921 unique players. In Experiment 1, one play with a negative reaction time was assumed to reflect a recording error and was removed.

In the online experiments, there were no restrictions on how many times each screen could be clicked before the target was found. It is therefore possible that in some cases, participants used a strategy where they randomly clicked the screen until they found the target by random chance. However, in most cases, participants clicked only once on each screen; only 5.2% of trials in Experiment 1 and 5.1% of trials in Experiment 2 had more than 5 clicks. Removing these trials from the analyses does not change the main conclusions (see Supplementary Material—Additional analyses 2), and Experiment 2B, where this shotgun strategy was not possible, shows the same trends (see Supplementary Materials, Experiment 2B).

## ANALYSIS

### Experiment 1—Is a specialist or generalist strategy more successful?

All statistical analyses for both experiments were carried out in R version 4.0.3 (“Bunnie-Wunnies Freak Out”) (R Core Team 2020). Cox proportional hazards survival models were fitted with the “survival” package (version 3.2.13; Therneau 2020). The initial model included fixed effects of condition (25%, 50%, or 75% blends of background A) and trial number. Participant number (defining each play) was included as a cluster term to account for individual differences in performance, including differences in the properties of the devices used to play the experiment. Model checking using the “cox.zph” function in the “survival” package (Therneau 2020) and diagnostic plots drawn with “ggcoxdiagnostics” in the “survminer” package (version 0.4.9; Kassambara and Kosinski 2019) indicated that the proportional hazards assumption was violated, and thus the condition variable was re-fitted as a time-dependent coefficient, using the “survSplit” function in the “survival” package. This function divided the data into three sections, determined by subjective visual inspection of crossing-over points in the survival curves: a section from 0 to 1.5 s, a section from 1.5 to 2.5 s, and then a “late” part of the trial after 2.5 s. The inclusion of a time-dependent coefficient ensured that the proportional hazards assumption was not broken for the variables of interest: the time splits were not chosen for theoretical reasons, and we did not expect the exact values to have specific meanings. During model interpretation, we therefore focus on describing the changes in survival across early and late parts of each trial. The model included only the final response on each trial (i.e., either the time of a successful hit, or the time out response; misses before the target was successfully found were not analyzed). To evaluate the importance of the condition variable, a second model was fitted with only the time-dependent strata and the trial number as fixed effects. This was compared with the full model using AIC (Sakamoto et al. 1986). Planned comparisons between conditions 25, 50, and 75 were investigated using hazard ratios (HRs) for each time split, where HR <1 indicates a lower detection risk (and thus higher survival probability) than the reference condition, while HR >1 indicates a higher detection risk (and thus lower survival). Differences between conditions were deemed significant when the 95% confidence intervals (CIs) for the HRs did not include HR = 1. Since our survival model could provide measures of relative detection risk only over specific time phases rather than across the whole experiment, we also calculated odds ratios (ORs), based on the total number of hits and time outs for each condition, to assess the relative benefit of each strategy overall. Pairwise ORs between conditions were calculated using the “oddsratio” function in the “epitools” package (Aragon 2020) and chi-square tests were used to test significance.

### Experiment 1—How do background properties affect the specialist generalist trade-off?

Each target was displayed on two different backgrounds. To assess how the difference between these backgrounds affected camouflage efficacy for the generalist and specialist conditions, we first compared the backgrounds in each pair using several metrics. A color difference metric was calculated by taking the Euclidean distance between the average L*a*b* values of each background, calculated from the RGB values using a custom-written MATLAB script. A pattern difference metric was taken using granularity analysis, carried out using custom plugins in Image J (Schneider et al. 2012): this involved filtering both backgrounds with a set of spatial frequencies (from 2 pixels to 2048 pixels, in steps of √2, giving 21 measurements for each image) then measuring the pattern energy (the standard deviation of the filtered image) at each frequency band for each background, and finally calculating the absolute difference in pattern energy between the two backgrounds by comparing their granularity curves (Troscianko and Stevens 2015). A luminance metric was calculated in a similar manner, summing the differences in the number of pixels in each luminance bin (32 bins from 0% to 100%) for both backgrounds (Troscianko and Stevens 2015). Finally, to create one overall metric of “background difference” (multidimensional pattern space or MDPS) for each pair, we took the Euclidean distance between two points in three-dimensional space, defined by the three difference variables (color, pattern, and luminance). Each difference metric was standardized by expressing each as a proportion of the maximum value for that metric (Spottiswoode and Stevens 2011).

The importance of each difference metric was assessed using a Cox proportional hazards model, with the quantitative difference between backgrounds added as a fixed effect, interacting with time-dependent condition. To test the relevance of each difference metric to the success of camouflage strategies, the full models were compared with a reduced model without any difference metric using AIC (Sakamoto et al. 1986).

### Experiment 2—Do generalists perform better when found on more than two backgrounds?

Similar to Experiment 1, results were initially analyzed using survival models including trial number and target condition (S, G2, and G4) as fixed effects, as well as distance between the target and the center of the screen, with participant number as a cluster term. Model diagnostics suggested that the proportional hazards assumption was violated, so a survival model was re-fitted with condition as a time-varying coefficient. Based on visual inspection of crossing points between the survival curves for each condition, the length of each trial was split into six phases: 0–0.6, 0.6–1.2, 1.2–1.4, 1.4–2, 2–10.5, and 10.5–15 s. As in Experiment 1, these time splits were selected to improve model diagnostics, and were not expected to have specific meanings in terms of participant behavior, so our interpretation again focused on broader trends in HRs early and late in the search phase. Diagnostic plots suggested that the only remaining factor causing substantial deviations from the assumption was the distance between the target and the center of the screen, so the final model was stratified by distance from the center; to that end, distance was transformed into a categorical variable with four levels corresponding to quartiles. The overall effects of condition and trial number were assessed using AIC. As in Experiment 1, only the final click on each slide was included in the analyses, planned comparisons between conditions S, G2, and G4 were investigated using HRs for each time split, and ORs were calculated to estimate the relative success of each strategy across the entire experiment.

## RESULTS

### Experiment 1—Is a specialist or generalist strategy more successful?

**Figure 3:**
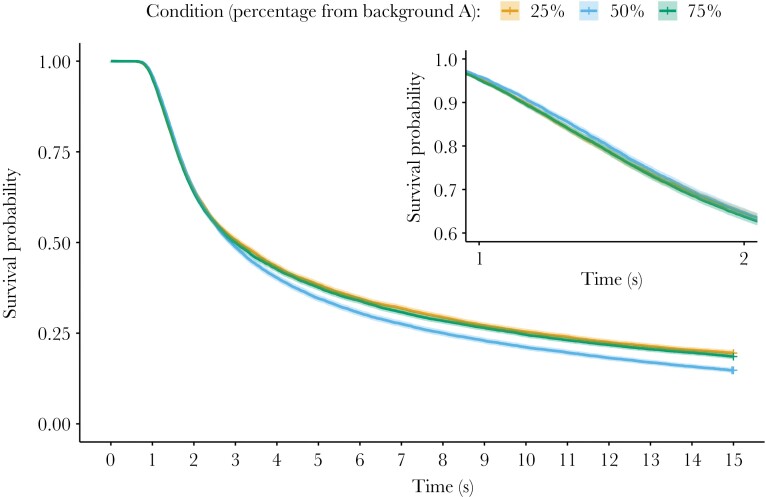
Survival probability for specialist and generalist targets over time in Experiment 1. Specialist targets are either more similar to background A (75% from background A) or background B (25% from background A), and would be expected to have equivalent survival. Generalist targets (50% condition) should be equally well matched on both backgrounds A and B. Crosses indicate censored (time out) data, and the shaded line indicates the 95% CI. The inset shows a zoomed-in section of the main curve, between 1 and 2 s, in order to highlight the crossover effect (see also the Supplementary Materials for a graph showing the summarized data for this time period for each condition).

We assessed the success of the different target strategies by considering their survival probability across both background types (i.e., all specialist targets were presented on both their “matched” and “mismatched” backgrounds). As expected, the survival probability is approximately equivalent for the two more specialist conditions (25% and 75% from background A, see Figure 3). ORs provide a sense of the overall success of each strategy over the whole experiment and find there is no difference between the two specialist strategies (OR = 0.943, 95% CI = 0.885–1.004 for survival of 75% targets relative to the 25% group; χ^2^ test, *P* = 0.0678), but that more generalist targets do not perform as well (OR = 0.717, 95% CI = 0.671–0.766 for survival of 50% targets relative to the 25% group; χ^2^ test, *P* < 0.001).

Survival analyses with time-stratified Cox proportional hazards models reveal that the relative success of the different conditions varies with time, as there is a significant interaction between condition and time in these data (ΔAIC of full model compared with simplified model = −137). The effect appears to be driven by changes in the relative success of generalist targets (condition 50%), which show approximately 7% higher survival than specialist targets for the initial 0- to 1.5-s time period but then equivalent or lower survival as each search trial progressed (HRs indicate approximately 20% higher survival for specialists in the latter stages; see Supplementary Table 1). There is also a small effect of trial number, with participants getting faster as the experiment progresses (HR for trial number = 1.015, 95% CI = 1.012–1.018; ΔAIC of full model compared with simplified model lacking trial number = −79).

Specialist targets can also be described as either matched (i.e., when presented on the background they are most similar to) or mismatched (i.e., presented on the other background). As expected, mismatched specialists perform most poorly and matched specialists perform the best, with generalists falling in-between. Mismatched specialists are much more likely to be detected at the beginning of a trial but perform more similar to the other categories later on, whereas the matched specialists are much more likely not to be found even by the end of the trial (see Supplementary Material—Additional analyses 3).

### How do background properties affect the specialist generalist trade-off?

**Figure 4:**
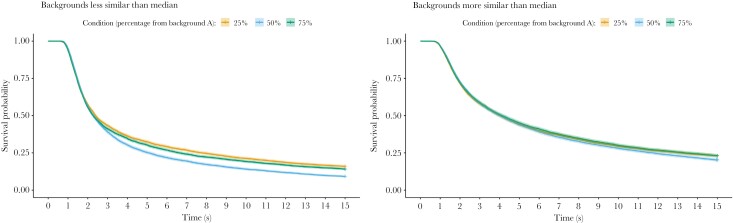
Survival probability of specialist and generalist targets over time in Experiment 1, split into cases where the background pairs are less similar than the median difference (left) or more similar than the median difference (right). Specialist targets are either more similar to background A (75% from background A) or background B (25% from background A), and would be expected to have equivalent survival. Generalist targets (50% condition) should be equally well matched on both A and B. Crosses indicate censored (time out) data, and the shaded line indicates the 95% CI.

For visualization purposes, we can split the dataset into cases where the similarity of the backgrounds is more or less than the median similarity (Figure 4). Where the backgrounds are less similar, there is a strong drop off in survival for the generalist strategy at the late time stages, while this decline is much less apparent where the backgrounds are more similar.

There is a significant effect of the interaction between time-varying effects of condition and the overall background difference metric (MDPS) and every other contrast metric, compared with the model with only time-dependent condition (Table 1). In the model including MDPS, in the early time period (0–1.5 s), as background difference increases, the initial disadvantage for the specialist conditions increases too (using the generalist 50% as the reference condition, 50–25*MDPS interaction: HR = 1.547, CI = 1.320–1.812; 50–75*MDPS interaction: HR = 1.763, CI = 1.489–2.087). However, this reverses in the later time period (2.5–15 s): for the majority of the trial length, generalists do worse than specialists as the backgrounds become more different (50–25*MDPS interaction: HR = 0.589, CI = 0.512–0.679; 50–75*MDPS interaction: HR = 0.671, CI = 0.582–0.774).

**Table 1:** AIC values for models including an interaction between target condition and several metrics of difference between pairs of backgrounds

Model specification	AIC	ΔAIC to best model
Null (condition only)	607 578.6	2077.8
Condition × Pattern difference	606 608.8	1108.1
Condition × Color difference	606 249.9	749.2
Condition × Luminance difference	605 980.2	479.4
Condition × MDPS	*605 500.7*	—

The full model had a concordance value (goodness-of-fit) of 0.584 (Wald test = 2016 on 21 df, *P* < 0.001).

italics indicate the AIC value of the best model.

According to model comparisons using AIC (Table 1), the overall measure of background difference (MDPS) best explains our results; if this is broken down into different visual features, the luminance match between backgrounds is the most powerful variable for predicting the effect of background difference on target detection, followed by color and finally pattern. In all cases, increasing the difference between the backgrounds decreases survival.

### Experiment 2—Do generalists perform better when found on more than two backgrounds?

**Figure 5:**
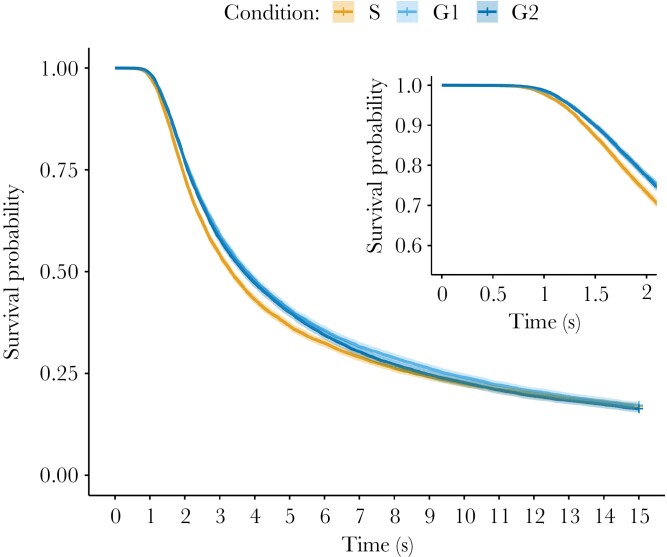
Survival probability of specialist (S) and G2 or G4 generalist targets over time in Experiment 2, with a zoomed-in section over the first 2 s of searching (inset), to show the differences in this time period more clearly. Crosses indicate censored (time out) data, and the shaded line indicates the 95% CI.

When targets are displayed against four background types there is no difference in overall detection probability between specialist and generalist targets (OR = 1.014, 95% CI = 0.953–1.078, χ^2^ test, *P* = 0.661, and OR = 0.958, 95% CI = 0.900–1.019, χ^2^ test, *P* = 0.174, for survival of G2 and G4, respectively, relative to S), or between G2 and G4 generalists (OR = 0.945, 95% CI = 0.888–1.005, χ^2^ test, *P* = 0.072 for survival of G4 relative to G2). However, time-stratified survival analyses once again reveal a significant, time-dependent, effect of target condition on survival probability (ΔAIC of full model compared with simplified model = −102.8; Figure 5). This seems to be driven by a shift in the relative success of specialist versus generalists strategies over the course of the time trial, with specialists (S) having equivalent or lower survival than generalists (G2 and G4) for most of the duration of the trials, then improving to perform better than generalists in the final stage (10.5–15 s; Supplementary Table 2). G2 and G4 generalists perform similar to each other over most time splits, with only a single phase in which G2 targets have higher survival, and one in which G4 targets survive better (Supplementary Table 2). As in Experiment 1, there is also a small effect of trial number, with detection risk increasing slightly as participants progress through the task (ΔAIC of full model compared with simplified model = −122.4, HR = 1.006, 95% CI = 1.004–1.007).

## DISCUSSION

Our experiments tested the relative benefits of specialist and generalist background-matching strategies for prey found on multiple backgrounds, where both the targets and backgrounds consist of complex naturalistic patterns, varying in aspects of their visual appearance. In our first experiment, we followed previous studies in which targets were displayed on two background types (Merilaita et al. 2001; Bond and Kamil 2006; Sherratt et al. 2007; Toh and Todd 2017). In the second, we expanded the number of backgrounds from two to four. We found that specialist targets outperformed generalists overall when displayed against two background types, but that when targets were shown against four background types, both strategies were equally successful over the entire experiment. In the latter scenario, generalists designed to be equally similar to all four background types (G4) and generalists composed from images of two background types (G2) performed similarly. Thus, some degree of imperfect camouflage can be adaptive, maintaining survival when prey occur on many different background types. Moreover, whether targets were seen against two or four background types, the effect of target condition on survival was time dependent within each target presentation at the population level, such that the population of specialists initially experienced higher mortality, but performed similar to or better than generalists in the final stages of each search trial.

Our study is the first to consider the effect on the performance of specialist and generalist targets of having more than two background types available. This more closely mimics natural situations, given that animals might not only travel between multiple distinct patches for various purposes, but may also have to contend with naturally heterogeneous backgrounds, varying both in space and time (Endler 1978, 1984; Baling et al. 2020). In Experiment 2, where targets were encountered on four different background types, we found a similar time-varying effect of strategy on survival to the two-background scenario in Experiment 1, where, on average, the population of specialists initially suffers a greater detection risk in the first few seconds of search, but later outperform generalists. However, the specialist disadvantage was much larger in the experiments across four backgrounds, in both the magnitude of difference in detection risk and the duration of the disadvantage, lasting approximately two thirds of each search trial. As a result, although specialists still outperformed generalists at the end of each trial, there was no overall difference in the benefits of specialist versus generalist strategies when they were viewed against more than two backgrounds. This pattern of time dependence makes intuitive sense and is supported by analyses of specialist performance against matched or mismatched backgrounds: poorly matched specialist targets on the wrong background may be found almost immediately, but specialists on their matched background are very difficult to detect, while generalist targets are of intermediate difficulty. Across multiple background types, specialists will be on their matched backgrounds on a smaller proportion of trials, so we would expect the early specialist disadvantage to be larger. With our targets and backgrounds, this ultimately led to equivalent survival for both strategies. Experiments 1 and 2 also differ in the degree of specialism of the specialist targets: in Experiment 1, specialist targets are biased blended images, consisting of 75% of one background type and 25% of another, while in Experiment 2, specialist targets are selected from a single background type (resemble it 100%). Yet, Experiment 1B (Supplementary Material, Experiment 1B), replicating Experiment 1 with a full specialist condition (100% from backgrounds A or B), shows that full specialists still outperform generalists in a two-background scenario, suggesting that the increase in number of background types is the key difference explaining the contrasting results of Experiments 1 and 2.

In both experiments, the time-dependent shift in the performance of specialists suggests that the risk for generalist and specialist background-matching prey is not uniform across time (although it should be noted that this is not a time-dependent effect for an individual, but is rather an emergent property over a population, or over the lifetime of an individual as it moves across habitats). HRs suggest that, in Experiment 1, specialists suffer 7% lower survival than generalists in the first 1.5 s of each search trial, then improve to outperform generalists by almost 20% from 2.5 s to the end of the trial. These differences in survival probability are relatively small, but are of a similar order of magnitude to those found in other work testing predation risk for camouflaged prey, such as in laboratory trials with seahorses attacking prawn color morphs on different backgrounds (Duarte et al. 2018), field tests of avian predation on peppered moth models (Walton and Stevens 2018) and detection of crab targets by human “predators” on screens (Troscianko et al. 2021). The initial disadvantage for specialists also worsens when targets are shown against four rather than two background types, suggesting that this trend could be especially relevant in patchy natural environments. In natural systems, these results could suggest that selection for habitat preference for specialists may be stronger in multi-background environments, although the current experiments cannot test this idea directly, as they assume targets are found equally often on all background types.

As generalists are harder to find, on average, in the first few seconds of searching, a cursory search may leave them undetected. Thus, predator behavior might affect the relative success of imperfect background matching, due to differences in predator searching strategy, which in turn might depend on motivation, attention, and cognitive abilities (Karpestam et al. 2014; Galloway et al. 2020). At a population level, generalist prey targets may be better suited to surviving encounters with predators likely to devote only a small amount of time and effort to searching. In natural situations, predators may have limited time and attention to devote to searching, as they balance foraging effort with other behaviors such as vigilance (Dukas 2002), while within foraging itself, trade-offs between exploiting a given patch or exploring new areas that could be more productive (Mehlhorn et al. 2015) may cause predators to abandon a search, creating conditions in which generalists could escape intense scrutiny and be successful.

The two-background experiment (Experiment 1), pairing different sets of natural backgrounds, also provided an opportunity to test the effect of background difference on the success of imperfect camouflage. As expected from theoretical models (Merilaita et al. 1999; Houston et al. 2007), we found that the difference in survival probability between generalist and specialist targets was smaller when backgrounds were more similar to each other. In fact, increased difference between background types magnified both aspects of the time-dependent variation between specialist and generalist strategies. When displayed on backgrounds that were more different from each other, the population of specialist targets experienced both a greater disadvantage in the earliest phase of the trials, and a larger advantage in the later stages of each trial. Previous explicit tests of the impact of background difference on the success of imperfect camouflage have demonstrated that generalists do better when backgrounds are more similar in terms of pattern size (Merilaita et al. 2001; Bond and Kamil 2006; Toh and Todd 2017). However, the effects of background differences in other visual properties have never previously been systematically tested in the context of imperfect camouflage. Experiments testing the effect of similarity between targets and their backgrounds on target detection have shown that many other features, such as pattern shape (Merilaita and Dimitrova 2014), color (Fennell et al. 2019), and luminance (Sherratt et al. 2007) are relevant. One recent study also considered multiple background properties in this context, concluding that matching the modal characteristics of a scene in both color and pattern minimizes detection risk (Michalis et al. 2017). Measures of background difference in terms of pattern, luminance, and color all had a significant effect on the relative success of generalist versus specialist targets in Experiment 1. However, excluding the multidimensional measure of background difference, luminance contrast between backgrounds was the most powerful predictor of target detection risk, followed by color. Despite the focus on pattern in tests of imperfect camouflage solutions (Merilaita et al. 2001; Bond and Kamil 2006; Toh and Todd 2017) and recent insights into the role of disruptive patterning for enhancing camouflage (Stevens and Cuthill 2006; Troscianko et al. 2018), we find pattern difference to be the least important background characteristic for successful imperfect camouflage.

The relative importance of luminance contrast between backgrounds in determining the effectiveness of imperfect strategies in this study echoes the results of a computer-based experiment where specialists and generalists performed similarly if the backgrounds differed in pattern, but specialists outperformed generalists when backgrounds varied in luminance (Sherratt et al. 2007). This suggests that human participants are more sensitive to contrasts in luminance than pattern, making it more difficult for a generalist target to evade detection if it is mismatched in luminance, at least when detecting targets on a computer screen (although recent work has shown good accordance between field- and computer-based results; Briolat et al. 2021). Luminance and saturation contrast between targets and backgrounds were also shown to predict detection times when volunteers searched for conspicuous targets (White et al. 2017). With the exception of Michalis et al. (2017), experiments testing the response of nonhuman animals to imperfect camouflage have been limited to targets varying only in pattern. Assessing the relative survival of prey specializing or generalizing with respect to background luminance and color would help determine the importance of these features in other systems, particularly for dichromatic predators, which may experience greater difficulty breaking camouflage based on color (Michalis et al. 2017).

Our results are limited by the fact that we tested only human predators, who may have quite different cognitive and perceptual abilities when compared with nonhuman predators. However, previous experiments have shown similar results for field trials with wild nonhuman predators compared with human detection experiments on screens (Stevens et al. 2013; Costello et al. 2020) or in the wild (Kjernsmo et al. 2020). Our experiments also do not reflect the three-dimensional environments that real animals are searched for and found in, although if anything, we would expect that larger and more complex setups would increase detection times and the differences seen between conditions (Troscianko et al. 2021). In addition, while our backgrounds cover a wider range of naturalistic images than used previously, they clearly remain limited compared with the full range of habitats available in nature. Recent work has also shown that background complexity can have important effects on the efficacy of generalist camouflage (Murali et al. 2021; Rowe et al. 2021): in the current study, we did not attempt to characterize background complexity, but how this interacts with target–background difference in our task would be an interesting question for future research.

Finally, our results suggest that variation in predator behavior might also be worthy of investigation, as it may affect the success of generalist and specialist strategies at the population level. Empirically, in experiments with humans, studies that record eye movements have helped provide more fine-scale measures of searching behavior, including better estimates of camouflage success (Lin et al. 2014) and evidence of how different strategies function, such as disruptive patterns affecting object recognition (Webster et al. 2013). It could also be valuable to manipulate the participants’ attention (e.g., by dividing attention between tasks, or offering multiple search targets; Dukas 2002) and motivation (e.g., by using food rewards of different values for animal experiments or monetary payments for human players, or by altering the frequency of targets) in order to determine how these traits affect the relative efficacy of different camouflage strategies. As predator search strategies may have important consequences for camouflage optimization, these should be considered more closely in future theoretical and empirical work.

## Supplementary Material

arac114_suppl_Supplementary_Materials

## Notes

Data Availability: Analyses reported in this article can be reproduced using the data provided by Hughes et al. (2022).
