# Supplementary material for: Varying benefits of generalist and specialist camouflage in two versus four background environments
Source: Behav Ecol. 2023 Mar 26;34(3):426–36. doi: 10.1093/beheco/arac114 (PMC10183209; doi:10.1093/beheco/arac114)
Supplement: arac114_suppl_Supplementary_Materials [file arac114_suppl_supplementary_materials.zip › RMarkdown_behavecol_final.html]

Reproducible code for manuscript: Time-dependent benefits of camouflage strategies on heterogeneous backgrounds


# Reproducible code for manuscript: Time-dependent benefits of camouflage strategies on heterogeneous backgrounds

#### Anna Hughes, Emmanuelle Briolat, Lina Arenas, Eric Liggins, Martin Stevens

#### Finalised 09/05/2022

## Load libraries

```
library(tidyverse)
library(car)
library(zoo)
library(survival)
library(survminer)
library(coxme)
library(patchwork)
library(grid)
library(gridExtra)
library(epitools)
library(rmarkdown)
library(broom)
library(pander)
library(knitr)
library(kableExtra)
```

Also load “multiplot” function from R cookbook (for plotting purposes)

---

## EXPERIMENT 1

### Load and prepare data

```
data <- read_csv('full_data_Expt1.csv') #survival data from the online experiment. 

RT_data_pair <- read_csv('pair_data_Expt1_nomobile.csv') #data for pairwise comparisons between backgrounds
```

Data for pairwise comparisons are split according to background similarity: background pairs more or less similar than median similarity.

```
median_MDPS <- median(RT_data_pair$MDPS)

RT_data_pair_top <- filter(RT_data_pair, MDPS > median_MDPS)
RT_data_pair_bottom <- filter(RT_data_pair, MDPS <= median_MDPS)

RT_data_pair_top_full <- gather(RT_data_pair_top, bgCond, RT, RT1, RT2)
RT_data_pair_bottom_full <- gather(RT_data_pair_bottom, bgCond, RT, RT1, RT2)

RT_data_pair_top_full <- RT_data_pair_top_full %>%
  mutate(Hit = 0, TrialNo = 0)

for (i in 1:nrow(RT_data_pair_top_full)){
  if (RT_data_pair_top_full$bgCond[i] == "RT1") {
    RT_data_pair_top_full$Hit[i] = RT_data_pair_top_full$Hit1[i]
    RT_data_pair_top_full$TrialNo[i] = RT_data_pair_top_full$TrialNo1[i]
  } else {
    RT_data_pair_top_full$Hit[i] = RT_data_pair_top_full$Hit2[i]
    RT_data_pair_top_full$TrialNo[i] = RT_data_pair_top_full$TrialNo2[i]
  }
}

RT_data_pair_bottom_full <- RT_data_pair_bottom_full %>%
  mutate(Hit = 0)

for (i in 1:nrow(RT_data_pair_bottom_full)){
  if (RT_data_pair_bottom_full$bgCond[i] == "RT1") {
    RT_data_pair_bottom_full$Hit[i] = RT_data_pair_bottom_full$Hit1[i]
    RT_data_pair_bottom_full$TrialNo[i] = RT_data_pair_bottom_full$TrialNo1[i]
  } else {
    RT_data_pair_bottom_full$Hit[i] = RT_data_pair_bottom_full$Hit2[i]
    RT_data_pair_bottom_full$TrialNo[i] = RT_data_pair_bottom_full$TrialNo2[i]
  }
}
```

Moth targets are defined as 25 or 75 % from Background 1 in data sheet - so targets in condition 75 are matched if shown on background 1, those in condition 25 are matched if shown on background 2.

```
data$Matched<-rep("Generalist", length(data$TrialNo))
for (i in 1:length(data$TrialNo)){
  if(data$Condition[i]==25){
    if(data$BgNum[i]==1){
      data$Matched[i]<-"Mismatched specialist"
    }
    else if (data$BgNum[i]==2){
      data$Matched[i]<-"Matched specialist"
    }
  }
  else if (data$Condition[i]==75){
    if(data$BgNum[i]==1){
      data$Matched[i]<-"Matched specialist"
    }
    else if (data$BgNum[i]==2){
      data$Matched[i]<-"Mismatched specialist"
    }
  }
}
```

To determine how many volunteers have taken part in the experiment:

```
data_nomobile <- filter(data, Device != "mobile")
data_naive <- filter(data_nomobile,PlayedBefore == "n")
```

3106 plays in total, excluding plays on mobile phones  
1717 unique players

To determine how many incorrect clicks occurred before participants found the target:

```
ClickSummary<-data_nomobile%>%
  group_by(TrialNo, Subject)%>%
  summarise (click_number=n())
```

```
## `summarise()` has grouped output by 'TrialNo'. You can override using the
## `.groups` argument.
```

```
#this data frame shows how many times each participant clicked for each trial

#How many trials with more than 5 clicks? 
ManyClicks<-ClickSummary%>%
  filter(click_number>5)
length(unique(ManyClicks$Subject))

PercentLargeClicks<-100*length(ManyClicks$TrialNo)/length(ClickSummary$TrialNo)
```

```
hist(ClickSummary$click_number)
  #this plot shows most targets are found with the first click; only a very small number of participants click more than 5 times, suggesting they do not generally use a scatter-gun approach to finding the target
```

The median number of clicks per target is 1 (mean = 1.7576733), so in most cases, participants only click when they spot the target. However, a small number of trials include larger numbers of clicks: the maximum number of clicks for a single trial is 41, but only 5.1861988 % of trials have over 5 clicks.

### Survival analyses

#### Prepare data for survival analyses

```
# Choose only the last row (final click)
survival_data <- data %>%
  group_by(Subject,TrialNo) %>%
  slice(n()) %>%
  ungroup()

# Replace NAs with full search time (15s)
survival_data$Time <- survival_data$Time %>% replace_na(15000)

# Divide time by 1000 to get it in s rather than ms
survival_data$Time <- survival_data$Time/1000

# Filter out glitchy data & mobile data
survival_data <- survival_data %>%
  filter(Device != "mobile") %>%
  filter(Time > 0) 

# Make condition a factor
survival_data$fCondition <- factor(survival_data$Condition)
survival_data$fBackground <- factor(survival_data$Background)
survival_data$fMoth <- factor(survival_data$Moth)
survival_data$fSubject <- factor(survival_data$Subject)
survival_data$fBackgroundType <- factor(survival_data$BackgroundType)

# Data for odds ratio
survival_data_OR <- survival_data %>%
  group_by(Condition) %>%
  summarise(Total = n(), Total_hits = sum(Hit))
```

#### Survival models

The survival model initially included trial number and condition, clustered by subject (participant).  
However, the proportional hazards assumption was broken in this model, so a new model was re-fitted with condition as a time-dependent coefficient. With this, the proportional hazards assumptions are met.

```
#Basic survival model - assumptions not met
assumption_fit <- coxph(Surv(Time, Hit) ~ fCondition + TrialNo, cluster =fSubject, data = survival_data)
summary(assumption_fit)
```

```
## Call:
## coxph(formula = Surv(Time, Hit) ~ fCondition + TrialNo, data = survival_data, 
##     cluster = fSubject)
## 
##   n= 37271, number of events= 30705 
## 
##                  coef exp(coef) se(coef) robust se     z Pr(>|z|)    
## fCondition50 0.104004  1.109605 0.013951  0.012395 8.391   <2e-16 ***
## fCondition75 0.025030  1.025346 0.014101  0.012950 1.933   0.0532 .  
## TrialNo      0.014673  1.014781 0.001640  0.001628 9.015   <2e-16 ***
## ---
## Signif. codes:  0 '***' 0.001 '**' 0.01 '*' 0.05 '.' 0.1 ' ' 1
## 
##              exp(coef) exp(-coef) lower .95 upper .95
## fCondition50     1.110     0.9012    1.0830     1.137
## fCondition75     1.025     0.9753    0.9996     1.052
## TrialNo          1.015     0.9854    1.0115     1.018
## 
## Concordance= 0.515  (se = 0.002 )
## Likelihood ratio test= 140.3  on 3 df,   p=<2e-16
## Wald test            = 152.2  on 3 df,   p=<2e-16
## Score (logrank) test = 140.8  on 3 df,   p=<2e-16,   Robust = 145.3  p=<2e-16
## 
##   (Note: the likelihood ratio and score tests assume independence of
##      observations within a cluster, the Wald and robust score tests do not).
```

```
cox.zph(assumption_fit)
```

```
##               chisq df      p
## fCondition 86.14998  2 <2e-16
## TrialNo     0.00229  1   0.96
## GLOBAL     86.15070  3 <2e-16
```

```
# Use survSplit to fit a time-dependent condition variable
fit_split_data <- survSplit(Surv(Time, Hit) ~ fCondition + fSubject + TrialNo,
                            data = survival_data, cut = c(1.5, 2.5), episode = "time_group")

fit_split <- coxph(Surv(tstart, Time, Hit) ~ fCondition:strata(time_group) + TrialNo, cluster = fSubject, data = fit_split_data)
summary(fit_split)
```

```
## Call:
## coxph(formula = Surv(tstart, Time, Hit) ~ fCondition:strata(time_group) + 
##     TrialNo, data = fit_split_data, cluster = fSubject)
## 
##   n= 87303, number of events= 30705 
## 
##                                                  coef exp(coef)  se(coef)
## TrialNo                                      0.014751  1.014860  0.001640
## fCondition25:strata(time_group)time_group=1 -0.005830  0.994187  0.027222
## fCondition50:strata(time_group)time_group=1 -0.074063  0.928613  0.027613
## fCondition75:strata(time_group)time_group=1        NA        NA  0.000000
## fCondition25:strata(time_group)time_group=2 -0.022385  0.977863  0.026686
## fCondition50:strata(time_group)time_group=2  0.049231  1.050463  0.026193
## fCondition75:strata(time_group)time_group=2        NA        NA  0.000000
## fCondition25:strata(time_group)time_group=3 -0.037202  0.963481  0.020962
## fCondition50:strata(time_group)time_group=3  0.182816  1.200593  0.020425
## fCondition75:strata(time_group)time_group=3        NA        NA  0.000000
##                                             robust se      z Pr(>|z|)    
## TrialNo                                      0.001629  9.057  < 2e-16 ***
## fCondition25:strata(time_group)time_group=1  0.025824 -0.226  0.82138    
## fCondition50:strata(time_group)time_group=1  0.025747 -2.877  0.00402 ** 
## fCondition75:strata(time_group)time_group=1  0.000000     NA       NA    
## fCondition25:strata(time_group)time_group=2  0.026333 -0.850  0.39527    
## fCondition50:strata(time_group)time_group=2  0.025461  1.934  0.05316 .  
## fCondition75:strata(time_group)time_group=2  0.000000     NA       NA    
## fCondition25:strata(time_group)time_group=3  0.020300 -1.833  0.06686 .  
## fCondition50:strata(time_group)time_group=3  0.020000  9.141  < 2e-16 ***
## fCondition75:strata(time_group)time_group=3  0.000000     NA       NA    
## ---
## Signif. codes:  0 '***' 0.001 '**' 0.01 '*' 0.05 '.' 0.1 ' ' 1
## 
##                                             exp(coef) exp(-coef) lower .95
## TrialNo                                        1.0149     0.9854    1.0116
## fCondition25:strata(time_group)time_group=1    0.9942     1.0058    0.9451
## fCondition50:strata(time_group)time_group=1    0.9286     1.0769    0.8829
## fCondition75:strata(time_group)time_group=1        NA         NA        NA
## fCondition25:strata(time_group)time_group=2    0.9779     1.0226    0.9287
## fCondition50:strata(time_group)time_group=2    1.0505     0.9520    0.9993
## fCondition75:strata(time_group)time_group=2        NA         NA        NA
## fCondition25:strata(time_group)time_group=3    0.9635     1.0379    0.9259
## fCondition50:strata(time_group)time_group=3    1.2006     0.8329    1.1544
## fCondition75:strata(time_group)time_group=3        NA         NA        NA
##                                             upper .95
## TrialNo                                        1.0181
## fCondition25:strata(time_group)time_group=1    1.0458
## fCondition50:strata(time_group)time_group=1    0.9767
## fCondition75:strata(time_group)time_group=1        NA
## fCondition25:strata(time_group)time_group=2    1.0297
## fCondition50:strata(time_group)time_group=2    1.1042
## fCondition75:strata(time_group)time_group=2        NA
## fCondition25:strata(time_group)time_group=3    1.0026
## fCondition50:strata(time_group)time_group=3    1.2486
## fCondition75:strata(time_group)time_group=3        NA
## 
## Concordance= 0.522  (se = 0.002 )
## Likelihood ratio test= 228.3  on 7 df,   p=<2e-16
## Wald test            = 233.9  on 7 df,   p=<2e-16
## Score (logrank) test = 231.1  on 7 df,   p=<2e-16,   Robust = 216.7  p=<2e-16
## 
##   (Note: the likelihood ratio and score tests assume independence of
##      observations within a cluster, the Wald and robust score tests do not).
```

```
cox.zph(fit_split)
```

```
##                                  chisq df    p
## TrialNo                        0.00112  1 0.97
## fCondition:strata(time_group) 10.53464  6 0.10
## GLOBAL                        10.53579  7 0.16
```

```
ggcoxdiagnostics(fit_split,type="schoenfeld")
```

Relevelling the model allows extraction of hazard ratios comparing all conditions, in all time splits.

```
# Relevel so condition 50 is the reference level (this works because it's using the last group as reference)
fit_split_data$fCondition <- relevel(fit_split_data$fCondition, "75")
fit_split <- coxph(Surv(tstart, Time, Hit) ~ fCondition:strata(time_group) + TrialNo, cluster = fSubject, data = fit_split_data)
summary(fit_split)

# Relevel so condition 25 is the reference level
fit_split_data$fCondition <- relevel(fit_split_data$fCondition, "50")
fit_split <- coxph(Surv(tstart, Time, Hit) ~ fCondition:strata(time_group) + TrialNo, cluster = fSubject, data = fit_split_data)
summary(fit_split)

# Null model to test effect of condition
fit_split_null <- coxph(Surv(tstart, Time, Hit) ~ TrialNo, cluster = fSubject,
                        data = fit_split_data)


# Null model to test effect of trial number
fit_split_null2 <- coxph(Surv(tstart, Time, Hit) ~ fCondition:strata(time_group), cluster = fSubject, data = fit_split_data)
```

Both trial number and condition (stratified by time) improve survival.

| Model | AIC |
| --- | --- |
| Full | 6.0757856^{5} |
| Trial only | 6.0771513^{5} |
| Condition only | 6.0765752^{5} |

### Plot survival curves for Experiment 1

```
# Setting up for graph 
graph_fit <- survfit(Surv(Time, Hit) ~ fCondition, data = survival_data, cluster = fSubject, type = "kaplan-meier")

# Plotting a survival curve
main_plot <- ggsurvplot(graph_fit, data = survival_data, conf.int = TRUE, pval = FALSE, break.time.by = 1,
                        legend.title = "Condition (percentage from Background A):",
                        legend.labs = c("25%", "50%", "75%"), 
                        palette = c("#E69F00", "#56B4E9", "#009E73"), font.x = 24, font.y = 24, 
                        font.tickslab = 20, font.legend = 16,
                        xlab = "Time (s)")
zoomed_plot <- ggsurvplot(graph_fit, data = survival_data, conf.int = TRUE, pval = FALSE, break.time.by = 1,
                          ylim = c(0.6,1), xlim = c(1,2),
                          legend.title = "Condition (percentage from Background A):",
                          legend.labs = c("25%", "50%", "75%"), 
                          palette = c("#E69F00", "#56B4E9", "#009E73"), font.x = 18, font.y = 18, 
                          font.tickslab = 16,font.legend = 16,
                          xlab = "Time (s)")

p_full <- main_plot$plot + 
  annotation_custom(grob = ggplotGrob(zoomed_plot$plot+theme(legend.position = "none")),
                    xmin = 7,xmax=Inf,ymin = .35,ymax = Inf)
p_full
```

```
# to save for paper
ggsave("Fig3.tiff", device='tiff', dpi=300)
```

**Figure 3** Survival probability for specialist (25/75%) and generalist (50%) targets in Experiment 1.

### Odds ratios

These provide a sense of survival for different conditions overall.  
Data for odds ratio calculations:

Number of targets and hits per condition in Experiment 1

| Condition | Total | Total\_hits |
| --- | --- | --- |
| 25 | 12424 | 10003 |
| 50 | 12423 | 10586 |
| 75 | 12424 | 10116 |

```
condition <- c("25", "50", "75")
hit <- c("Yes", "No")
dat1 <- matrix(c(10003, 2421, 10586, 1837, 10116, 2308), nrow = 3, ncol = 2, byrow = TRUE)
dimnames(dat1) <- list("Condition" = condition, "Hit" = hit)
or_expt1<-oddsratio(dat1)
```

Results from odds ratio calculation (relative to 25% specialists):

Odds ratios relative to 25% specialists

|  | estimate | lower | upper |
| --- | --- | --- | --- |
| 25 | 1.000 | NA | NA |
| 50 | 0.717 | 0.671 | 0.766 |
| 75 | 0.943 | 0.885 | 1.004 |

Statistical tests for odds ratios

|  | midp.exact | fisher.exact | chi.square |
| --- | --- | --- | --- |
| 25 | NA | NA | NA |
| 50 | 0.0000 | 0.0000 | 0.0000 |
| 75 | 0.0679 | 0.0703 | 0.0678 |

### Effects of background similarity

#### Prepare data for comparisons between background pairs

```
## Backgrounds that are more different than the median

# Replace NAs, sort out time, filter out any glitchy data, make condition a factor
RT_data_pair_top_full$RT <- RT_data_pair_top_full$RT %>% replace_na(15000)
RT_data_pair_top_full$RT <- RT_data_pair_top_full$RT/1000

RT_data_pair_top_full <- RT_data_pair_top_full %>%
  filter(RT > 0) 

RT_data_pair_top_full$fCondition <- factor(RT_data_pair_top_full$condition)
RT_data_pair_top_full$fSubject <- factor(RT_data_pair_top_full$Subject)

## Backgrounds that are less different than the median

# Replace NAs, sort out time, filter out any glitchy data, make condition a factor
RT_data_pair_bottom_full$RT <- RT_data_pair_bottom_full$RT %>% replace_na(15000)
RT_data_pair_bottom_full$RT <- RT_data_pair_bottom_full$RT/1000

RT_data_pair_bottom_full <- RT_data_pair_bottom_full %>%
  filter(RT > 0)

RT_data_pair_bottom_full$fCondition <- factor(RT_data_pair_bottom_full$condition)
RT_data_pair_bottom_full$fSubject <- factor(RT_data_pair_bottom_full$Subject)
```

#### Plot curves for backgrounds more or less similar to median similarity

```
# Survival curve fit for high and low similarity
fit_top <- survfit(Surv(RT, Hit) ~ fCondition, data = RT_data_pair_top_full, cluster = fSubject,
                   type = "kaplan-meier")
fit_bottom <- survfit(Surv(RT, Hit) ~ fCondition, data = RT_data_pair_bottom_full, cluster = fSubject, 
                      type = "kaplan-meier")

# Plot
plot_top <- ggsurvplot(fit_top, data = RT_data_pair_top_full, conf.int = TRUE, pval = FALSE, break.time.by = 1,  
                       legend.title = "Condition (percentage from Background A):",
                       legend.labs = c("25%", "50%", "75%"), 
                       palette = c("#E69F00", "#56B4E9", "#009E73"), font.x = 16, font.y = 16, 
                       font.tickslab = 14, font.legend = 8, font.title = 14,
                       title = "a. Backgrounds less similar than median", xlab = "Time (s)")

#plot_top

# to save for paper
#ggsave("Fig4_a.tiff", device='tiff', dpi=300)

plot_bottom <- ggsurvplot(fit_bottom, data = RT_data_pair_bottom_full, conf.int = TRUE, pval = FALSE, 
                          break.time.by = 1,  
                          legend.title = "Condition (percentage from Background A):",
                          legend.labs = c("25%", "50%", "75%"), 
                          palette = c("#E69F00", "#56B4E9", "#009E73"), font.x = 16, font.y = 16, 
                          font.tickslab = 14, font.legend = 8,font.title = 14,
                          title = "b. Backgrounds more similar than median", xlab = "Time (s)")

#plot_bottom

# to save for paper
#ggsave("Fig4_b.tiff", device='tiff', dpi=300)

# Plot both curves together
multiplot(plot_top$plot, plot_bottom$plot, cols=2, restoreConsole = TRUE)
```

```
## [1] TRUE
```

**Figure 4** Survival probability for specialist (25/75%) and generalist (50%) targets in Experiment 1, split by similarity between background pairs.

#### Survival analyses with background similarity

Survival models initially included trial number, condition and a measure of background similarity, clustered by subject (participant).  
MDPS is an overall measure of background similarity, including luminance, colour and pattern.  
As above, the proportional hazards assumption was broken, so new models fit condition as a time-dependent variable, improving diagnostics.

```
# add a marker for background similarity to help plot graphs
RT_data_pair_top_full <- add_column(RT_data_pair_top_full, background_similarity = "top")
RT_data_pair_bottom_full <- add_column(RT_data_pair_bottom_full, background_similarity = "bottom")

RT_data_pair_full <- rbind(RT_data_pair_top_full, RT_data_pair_bottom_full)
RT_data_pair_full$fCondition <- droplevels(RT_data_pair_full$fCondition)
RT_data_pair_full$fSubject <- factor(RT_data_pair_full$Subject)
RT_data_pair_full$fBackground_similarity <- factor(RT_data_pair_full$background_similarity)

# adding in quantiles
RT_data_pair_full$MDPSQuant <- ntile(RT_data_pair_full$MDPS, 4)
RT_data_pair_full$PatternQuant <- ntile(RT_data_pair_full$patternEnergyDiffStandard, 4)
RT_data_pair_full$LumQuant <- ntile(RT_data_pair_full$coverageDiffStandard, 4)
RT_data_pair_full$ColourQuant <- ntile(RT_data_pair_full$maxColourDiffStandard, 4)

# assumptions don't seem to be met with a simple survival model
# leaving out frailty for testing cox zph?
fit_full_coxph <- coxph(Surv(RT,Hit) ~ fCondition * MDPS + TrialNo, cluster = fSubject, data = RT_data_pair_full)
```

```
cox.zph(fit_full_coxph)
```

```
##                    chisq df      p
## fCondition      1.22e+02  2 <2e-16
## MDPS            3.41e+02  1 <2e-16
## TrialNo         1.55e-03  1   0.97
## fCondition:MDPS 1.03e+02  2 <2e-16
## GLOBAL          4.81e+02  6 <2e-16
```

```
# use survSplit to break up into separate time stages
full_split_data <- survSplit(Surv(RT, Hit) ~ fSubject + fCondition + MDPS + TrialNo,
                             data = RT_data_pair_full, cut = c(1.5,2.5), episode = "time_group")

full_split <- coxph(Surv(tstart, RT, Hit) ~ fCondition:strata(time_group) * MDPS + TrialNo, cluster =fSubject, data = full_split_data)
summary(full_split)
```

```
## Call:
## coxph(formula = Surv(tstart, RT, Hit) ~ fCondition:strata(time_group) * 
##     MDPS + TrialNo, data = full_split_data, cluster = fSubject)
## 
##   n= 87303, number of events= 30705 
## 
##                                                       coef exp(coef)  se(coef)
## MDPS                                              0.340177  1.405197  0.051540
## TrialNo                                           0.015766  1.015891  0.001638
## fCondition25:strata(time_group)time_group=1       0.102144  1.107543  0.077022
## fCondition50:strata(time_group)time_group=1      -0.002112  0.997890  0.078185
## fCondition75:strata(time_group)time_group=1             NA        NA  0.000000
## fCondition25:strata(time_group)time_group=2       0.029204  1.029634  0.071390
## fCondition50:strata(time_group)time_group=2      -0.040406  0.960400  0.070735
## fCondition75:strata(time_group)time_group=2             NA        NA  0.000000
## fCondition25:strata(time_group)time_group=3       0.047022  1.048145  0.051989
## fCondition50:strata(time_group)time_group=3      -0.068876  0.933443  0.051235
## fCondition75:strata(time_group)time_group=3             NA        NA  0.000000
## fCondition25:strata(time_group)time_group=1:MDPS  0.835498  2.305963  0.081638
## fCondition50:strata(time_group)time_group=1:MDPS  0.874825  2.398454  0.082655
## fCondition75:strata(time_group)time_group=1:MDPS  0.966101  2.627679  0.082286
## fCondition25:strata(time_group)time_group=2:MDPS  0.537591  1.711878  0.081259
## fCondition50:strata(time_group)time_group=2:MDPS  0.730214  2.075525  0.079608
## fCondition75:strata(time_group)time_group=2:MDPS  0.607255  1.835386  0.081827
## fCondition25:strata(time_group)time_group=3:MDPS -0.129938  0.878150  0.072068
## fCondition50:strata(time_group)time_group=3:MDPS  0.399236  1.490686  0.070552
## fCondition75:strata(time_group)time_group=3:MDPS        NA        NA  0.000000
##                                                  robust se      z Pr(>|z|)    
## MDPS                                              0.054624  6.228 4.74e-10 ***
## TrialNo                                           0.001632  9.663  < 2e-16 ***
## fCondition25:strata(time_group)time_group=1       0.079512  1.285    0.199    
## fCondition50:strata(time_group)time_group=1       0.084381 -0.025    0.980    
## fCondition75:strata(time_group)time_group=1       0.000000     NA       NA    
## fCondition25:strata(time_group)time_group=2       0.070824  0.412    0.680    
## fCondition50:strata(time_group)time_group=2       0.068702 -0.588    0.556    
## fCondition75:strata(time_group)time_group=2       0.000000     NA       NA    
## fCondition25:strata(time_group)time_group=3       0.053537  0.878    0.380    
## fCondition50:strata(time_group)time_group=3       0.051936 -1.326    0.185    
## fCondition75:strata(time_group)time_group=3       0.000000     NA       NA    
## fCondition25:strata(time_group)time_group=1:MDPS  0.084312  9.910  < 2e-16 ***
## fCondition50:strata(time_group)time_group=1:MDPS  0.090596  9.656  < 2e-16 ***
## fCondition75:strata(time_group)time_group=1:MDPS  0.087867 10.995  < 2e-16 ***
## fCondition25:strata(time_group)time_group=2:MDPS  0.082117  6.547 5.89e-11 ***
## fCondition50:strata(time_group)time_group=2:MDPS  0.082129  8.891  < 2e-16 ***
## fCondition75:strata(time_group)time_group=2:MDPS  0.082516  7.359 1.85e-13 ***
## fCondition25:strata(time_group)time_group=3:MDPS  0.075436 -1.722    0.085 .  
## fCondition50:strata(time_group)time_group=3:MDPS  0.072918  5.475 4.37e-08 ***
## fCondition75:strata(time_group)time_group=3:MDPS  0.000000     NA       NA    
## ---
## Signif. codes:  0 '***' 0.001 '**' 0.01 '*' 0.05 '.' 0.1 ' ' 1
## 
##                                                  exp(coef) exp(-coef) lower .95
## MDPS                                                1.4052     0.7116    1.2625
## TrialNo                                             1.0159     0.9844    1.0126
## fCondition25:strata(time_group)time_group=1         1.1075     0.9029    0.9477
## fCondition50:strata(time_group)time_group=1         0.9979     1.0021    0.8458
## fCondition75:strata(time_group)time_group=1             NA         NA        NA
## fCondition25:strata(time_group)time_group=2         1.0296     0.9712    0.8962
## fCondition50:strata(time_group)time_group=2         0.9604     1.0412    0.8394
## fCondition75:strata(time_group)time_group=2             NA         NA        NA
## fCondition25:strata(time_group)time_group=3         1.0481     0.9541    0.9437
## fCondition50:strata(time_group)time_group=3         0.9334     1.0713    0.8431
## fCondition75:strata(time_group)time_group=3             NA         NA        NA
## fCondition25:strata(time_group)time_group=1:MDPS    2.3060     0.4337    1.9547
## fCondition50:strata(time_group)time_group=1:MDPS    2.3985     0.4169    2.0082
## fCondition75:strata(time_group)time_group=1:MDPS    2.6277     0.3806    2.2120
## fCondition25:strata(time_group)time_group=2:MDPS    1.7119     0.5842    1.4574
## fCondition50:strata(time_group)time_group=2:MDPS    2.0755     0.4818    1.7669
## fCondition75:strata(time_group)time_group=2:MDPS    1.8354     0.5448    1.5613
## fCondition25:strata(time_group)time_group=3:MDPS    0.8781     1.1388    0.7575
## fCondition50:strata(time_group)time_group=3:MDPS    1.4907     0.6708    1.2922
## fCondition75:strata(time_group)time_group=3:MDPS        NA         NA        NA
##                                                  upper .95
## MDPS                                                 1.564
## TrialNo                                              1.019
## fCondition25:strata(time_group)time_group=1          1.294
## fCondition50:strata(time_group)time_group=1          1.177
## fCondition75:strata(time_group)time_group=1             NA
## fCondition25:strata(time_group)time_group=2          1.183
## fCondition50:strata(time_group)time_group=2          1.099
## fCondition75:strata(time_group)time_group=2             NA
## fCondition25:strata(time_group)time_group=3          1.164
## fCondition50:strata(time_group)time_group=3          1.033
## fCondition75:strata(time_group)time_group=3             NA
## fCondition25:strata(time_group)time_group=1:MDPS     2.720
## fCondition50:strata(time_group)time_group=1:MDPS     2.864
## fCondition75:strata(time_group)time_group=1:MDPS     3.122
## fCondition25:strata(time_group)time_group=2:MDPS     2.011
## fCondition50:strata(time_group)time_group=2:MDPS     2.438
## fCondition75:strata(time_group)time_group=2:MDPS     2.158
## fCondition25:strata(time_group)time_group=3:MDPS     1.018
## fCondition50:strata(time_group)time_group=3:MDPS     1.720
## fCondition75:strata(time_group)time_group=3:MDPS        NA
## 
## Concordance= 0.584  (se = 0.002 )
## Likelihood ratio test= 2324  on 16 df,   p=<2e-16
## Wald test            = 2012  on 16 df,   p=<2e-16
## Score (logrank) test = 2429  on 16 df,   p=<2e-16,   Robust = 1235  p=<2e-16
## 
##   (Note: the likelihood ratio and score tests assume independence of
##      observations within a cluster, the Wald and robust score tests do not).
```

```
AIC(full_split)
```

```
## [1] 605500.7
```

```
# condition only
full_split_condonly <- coxph(Surv(tstart, RT, Hit) ~ fCondition:strata(time_group) + TrialNo, cluster = fSubject, data = full_split_data)
AIC(full_split_condonly)
```

```
## [1] 607578.6
```

```
cox.zph(full_split) #
```

```
##                                      chisq df       p
## MDPS                               26.3049  1 2.9e-07
## TrialNo                             0.0137  1 0.90679
## fCondition:strata(time_group)      11.8558  6 0.06526
## fCondition:strata(time_group):MDPS 18.3295  8 0.01889
## GLOBAL                             44.4809 16 0.00017
```

```
ggcoxdiagnostics(full_split,type="schoenfeld") # deviations are very small on this type of plot?
```

```
# Relevel so condition 50 is the reference level
full_split_data$fCondition <- relevel(full_split_data$fCondition, "75")
full_split <- coxph(Surv(tstart, RT, Hit) ~ fCondition:strata(time_group) * MDPS + TrialNo, cluster = fSubject, data = full_split_data)
summary(full_split)
```

```
## Call:
## coxph(formula = Surv(tstart, RT, Hit) ~ fCondition:strata(time_group) * 
##     MDPS + TrialNo, data = full_split_data, cluster = fSubject)
## 
##   n= 87303, number of events= 30705 
## 
##                                                       coef exp(coef)  se(coef)
## MDPS                                              0.739413  2.094707  0.048214
## TrialNo                                           0.015766  1.015891  0.001638
## fCondition75:strata(time_group)time_group=1       0.002112  1.002114  0.078185
## fCondition25:strata(time_group)time_group=1       0.104256  1.109885  0.077217
## fCondition50:strata(time_group)time_group=1             NA        NA  0.000000
## fCondition75:strata(time_group)time_group=2       0.040406  1.041233  0.070735
## fCondition25:strata(time_group)time_group=2       0.069609  1.072089  0.070210
## fCondition50:strata(time_group)time_group=2             NA        NA  0.000000
## fCondition75:strata(time_group)time_group=3       0.068876  1.071303  0.051235
## fCondition25:strata(time_group)time_group=3       0.115898  1.122881  0.050592
## fCondition50:strata(time_group)time_group=3             NA        NA  0.000000
## fCondition75:strata(time_group)time_group=1:MDPS  0.566865  1.762732  0.080245
## fCondition25:strata(time_group)time_group=1:MDPS  0.436262  1.546914  0.079580
## fCondition50:strata(time_group)time_group=1:MDPS  0.475588  1.608961  0.080622
## fCondition75:strata(time_group)time_group=2:MDPS  0.208019  1.231236  0.079773
## fCondition25:strata(time_group)time_group=2:MDPS  0.138355  1.148383  0.079191
## fCondition50:strata(time_group)time_group=2:MDPS  0.330978  1.392330  0.077495
## fCondition75:strata(time_group)time_group=3:MDPS -0.399236  0.670832  0.070552
## fCondition25:strata(time_group)time_group=3:MDPS -0.529174  0.589091  0.069723
## fCondition50:strata(time_group)time_group=3:MDPS        NA        NA  0.000000
##                                                  robust se      z Pr(>|z|)    
## MDPS                                              0.049649 14.893  < 2e-16 ***
## TrialNo                                           0.001632  9.663  < 2e-16 ***
## fCondition75:strata(time_group)time_group=1       0.084381  0.025  0.98003    
## fCondition25:strata(time_group)time_group=1       0.080170  1.300  0.19345    
## fCondition50:strata(time_group)time_group=1       0.000000     NA       NA    
## fCondition75:strata(time_group)time_group=2       0.068702  0.588  0.55645    
## fCondition25:strata(time_group)time_group=2       0.069960  0.995  0.31974    
## fCondition50:strata(time_group)time_group=2       0.000000     NA       NA    
## fCondition75:strata(time_group)time_group=3       0.051936  1.326  0.18478    
## fCondition25:strata(time_group)time_group=3       0.050957  2.274  0.02294 *  
## fCondition50:strata(time_group)time_group=3       0.000000     NA       NA    
## fCondition75:strata(time_group)time_group=1:MDPS  0.086068  6.586 4.51e-11 ***
## fCondition25:strata(time_group)time_group=1:MDPS  0.080780  5.401 6.64e-08 ***
## fCondition50:strata(time_group)time_group=1:MDPS  0.084174  5.650 1.60e-08 ***
## fCondition75:strata(time_group)time_group=2:MDPS  0.079545  2.615  0.00892 ** 
## fCondition25:strata(time_group)time_group=2:MDPS  0.078849  1.755  0.07931 .  
## fCondition50:strata(time_group)time_group=2:MDPS  0.076820  4.309 1.64e-05 ***
## fCondition75:strata(time_group)time_group=3:MDPS  0.072918 -5.475 4.37e-08 ***
## fCondition25:strata(time_group)time_group=3:MDPS  0.072068 -7.343 2.09e-13 ***
## fCondition50:strata(time_group)time_group=3:MDPS  0.000000     NA       NA    
## ---
## Signif. codes:  0 '***' 0.001 '**' 0.01 '*' 0.05 '.' 0.1 ' ' 1
## 
##                                                  exp(coef) exp(-coef) lower .95
## MDPS                                                2.0947     0.4774    1.9005
## TrialNo                                             1.0159     0.9844    1.0126
## fCondition75:strata(time_group)time_group=1         1.0021     0.9979    0.8494
## fCondition25:strata(time_group)time_group=1         1.1099     0.9010    0.9485
## fCondition50:strata(time_group)time_group=1             NA         NA        NA
## fCondition75:strata(time_group)time_group=2         1.0412     0.9604    0.9101
## fCondition25:strata(time_group)time_group=2         1.0721     0.9328    0.9347
## fCondition50:strata(time_group)time_group=2             NA         NA        NA
## fCondition75:strata(time_group)time_group=3         1.0713     0.9334    0.9676
## fCondition25:strata(time_group)time_group=3         1.1229     0.8906    1.0162
## fCondition50:strata(time_group)time_group=3             NA         NA        NA
## fCondition75:strata(time_group)time_group=1:MDPS    1.7627     0.5673    1.4891
## fCondition25:strata(time_group)time_group=1:MDPS    1.5469     0.6464    1.3204
## fCondition50:strata(time_group)time_group=1:MDPS    1.6090     0.6215    1.3643
## fCondition75:strata(time_group)time_group=2:MDPS    1.2312     0.8122    1.0535
## fCondition25:strata(time_group)time_group=2:MDPS    1.1484     0.8708    0.9839
## fCondition50:strata(time_group)time_group=2:MDPS    1.3923     0.7182    1.1977
## fCondition75:strata(time_group)time_group=3:MDPS    0.6708     1.4907    0.5815
## fCondition25:strata(time_group)time_group=3:MDPS    0.5891     1.6975    0.5115
## fCondition50:strata(time_group)time_group=3:MDPS        NA         NA        NA
##                                                  upper .95
## MDPS                                                2.3088
## TrialNo                                             1.0191
## fCondition75:strata(time_group)time_group=1         1.1823
## fCondition25:strata(time_group)time_group=1         1.2987
## fCondition50:strata(time_group)time_group=1             NA
## fCondition75:strata(time_group)time_group=2         1.1913
## fCondition25:strata(time_group)time_group=2         1.2296
## fCondition50:strata(time_group)time_group=2             NA
## fCondition75:strata(time_group)time_group=3         1.1861
## fCondition25:strata(time_group)time_group=3         1.2408
## fCondition50:strata(time_group)time_group=3             NA
## fCondition75:strata(time_group)time_group=1:MDPS    2.0866
## fCondition25:strata(time_group)time_group=1:MDPS    1.8123
## fCondition50:strata(time_group)time_group=1:MDPS    1.8976
## fCondition75:strata(time_group)time_group=2:MDPS    1.4390
## fCondition25:strata(time_group)time_group=2:MDPS    1.3403
## fCondition50:strata(time_group)time_group=2:MDPS    1.6186
## fCondition75:strata(time_group)time_group=3:MDPS    0.7739
## fCondition25:strata(time_group)time_group=3:MDPS    0.6785
## fCondition50:strata(time_group)time_group=3:MDPS        NA
## 
## Concordance= 0.584  (se = 0.002 )
## Likelihood ratio test= 2324  on 16 df,   p=<2e-16
## Wald test            = 2012  on 16 df,   p=<2e-16
## Score (logrank) test = 2429  on 16 df,   p=<2e-16,   Robust = 1235  p=<2e-16
## 
##   (Note: the likelihood ratio and score tests assume independence of
##      observations within a cluster, the Wald and robust score tests do not).
```

### Importance of different contrast variables

Here we test the effects of different measures of background similarity: luminance contrast, colour contrast and pattern difference. All models show a significant effect of the condition:contrast metric interaction (and for all models, the proportional hazards assumption is respected for the key interaction).

```
# survSplit for different variables
variables_split_data <- survSplit(Surv(RT, Hit) ~ fCondition + fSubject + patternEnergyDiffStandard + coverageDiffStandard + maxColourDiffStandard + TrialNo, data = RT_data_pair_full, cut = c(1.5,2.5), episode = "time_group")

#pattern metric
fit_pattern <- coxph(Surv(tstart, RT, Hit) ~ fCondition:strata(time_group) * patternEnergyDiffStandard + TrialNo, cluster = fSubject, data = variables_split_data)
#cox.zph(fit_pattern)
#anova(fit_pattern, full_split_interaction)

#luminance metric
fit_lum <- coxph(Surv(tstart, RT, Hit) ~ fCondition:strata(time_group) * coverageDiffStandard + TrialNo, cluster = fSubject, data = variables_split_data)
#cox.zph(fit_lum)
#anova(fit_lum, full_split_interaction)

#colour metric
fit_colour <- coxph(Surv(tstart, RT, Hit) ~ fCondition:strata(time_group) * maxColourDiffStandard + TrialNo, cluster = fSubject, data = variables_split_data)
#cox.zph(fit_colour)
#anova(fit_colour, full_split_interaction)

#compare models
AIC1<-AIC(full_split_condonly)
AIC2<-AIC(fit_pattern) 
AIC3<-AIC(fit_lum) 
AIC4<-AIC(fit_colour) 
AIC5<-AIC(full_split)
```

Comparing models using AIC suggests that colour is the most powerful predictor of survival, followed by luminance, then pattern. All models including contrast metrics perform better than the null model, but less well than the model with MDPS:

| Model | AIC |
| --- | --- |
| Condition:MDPS | 6.0550074^{5} |
| Condition:luminance | 6.0598016^{5} |
| Condition:colour | 6.0624992^{5} |
| Condition:pattern | 6.0660883^{5} |
| Null (condition only) | 6.0757856^{5} |

### Supplementary analyses - plots splitting specialists into matched/mismatched types

Additional analyses, separating specialist targets into matched (75% A on background A; 75% B on background B), or mismatched (25% A on background A; 25% B on background B).

Plots of both survival curves and capture times show that matched specialists perform best, mismatched specialists have the worst survival, and generalists are intermediate.

```
survival_data$Matched<-as.factor(survival_data$Matched)
survival_data$Matched<-factor(survival_data$Matched, levels=c("Generalist","Matched specialist","Mismatched specialist"))

# Setting up for survival plot
graph_fit_match <- survfit(Surv(Time, Hit) ~ Matched, data = survival_data, type = "kaplan-meier")

# Plotting a survival curve
main_plot <- ggsurvplot(graph_fit_match, data = survival_data, conf.int = TRUE, 
                        pval = FALSE, break.time.by = 1,
                        legend.title = "Condition :",
                        legend.labs = c( "Generalist", "Matched Specialist","Mismatched Specialist"), 
                        palette = c("#56B4E9","#E69F00",  "#009E73"), 
                        font.x = 24, font.y = 24, 
                        font.tickslab = 20, font.legend = 16,
                        xlab = "Time (s)")
main_plot
```

**Supplementary Figure 4** Survival probability for specialist (matched/mismatched) and generalist targets in Experiment 1.

We can also plot time of capture for matched/mismatched specialists and generalists:

```
my_theme<-theme(panel.grid.major = element_blank(), panel.grid.minor = element_blank(),
panel.background = element_blank(), axis.line = element_line(colour = "black"), axis.text = element_text(size = 20),axis.title = element_text(size = 24),legend.text = element_text(size = 16), legend.title = element_text(size = 16))

ggplot(survival_data, aes(x=Time, fill=Matched)) + 
  geom_histogram(aes(y = stat(density)), position="dodge")+
  scale_y_continuous(labels = scales::percent_format(suffix="",accuracy=1), 
                     limits = c(0,0.7), expand = c(0, 0))+
   scale_x_continuous(expand = c(0, 0))+
   scale_fill_manual(name="Condition", values=c( "#56B4E9","#E69F00","#009E73"),
                     labels=c("Generalist", "Matched Specialist","Mismatched Specialist"))+
   xlab("Time (s)") + ylab("Targets captured (%)")+
   my_theme+ theme(legend.position="top")
```

**Supplementary Figure 5** Histogram of capture times for specialist (matched/mismatched) and generalist targets in Experiment 1.

### Supplementary analyses - repeat main analyses, excluding trials with over 5 clicks

```
#Step 1: remove plays where participants made > 5 clicks
ManyClicks$Subj_Trial<-paste(ManyClicks$Subject,ManyClicks$TrialNo,sep="_")
survival_data_repeat<-survival_data
survival_data_repeat$TooManyClicks<-rep("no",length(survival_data$TrialNo))
survival_data_repeat$Subj_Trial<-paste(survival_data_repeat$Subject,
                                       survival_data_repeat$TrialNo,sep="_")

for (i in 1:length(survival_data_repeat$TrialNo)){
  for(j in 1:length(ManyClicks$Subj_Trial)){
    if(survival_data_repeat$Subj_Trial[i]==ManyClicks$Subj_Trial[j]){
      survival_data_repeat$TooManyClicks[i]<-"yes"
    }
  }
  #print(i)
}

survival_data_repeat2<-survival_data_repeat%>%
  filter(TooManyClicks!="yes")#37271-1933=35338 values now

#Step 2: repeat survival analyses

#Basic survival model - assumptions not met
assumption_fit <- coxph(Surv(Time, Hit) ~ fCondition + TrialNo, cluster = fSubject, data = survival_data_repeat2)
cox.zph(assumption_fit)#assumption broken for condition
```

```
##              chisq df      p
## fCondition 87.1263  2 <2e-16
## TrialNo     0.0069  1   0.93
## GLOBAL     87.1337  3 <2e-16
```

```
# Use survSplit to fit a time-dependent condition variable - with same splits as for full dataset
fit_split_data_repeat <- survSplit(Surv(Time, Hit) ~ fCondition + fSubject + TrialNo,
                            data = survival_data_repeat2, cut = c(1.5,2.5), episode = "time_group")

fit_split_repeat <- coxph(Surv(tstart, Time, Hit) ~ fCondition:strata(time_group) + TrialNo, cluster = fSubject, data = fit_split_data_repeat)
summary(fit_split_repeat)
```

```
## Call:
## coxph(formula = Surv(tstart, Time, Hit) ~ fCondition:strata(time_group) + 
##     TrialNo, data = fit_split_data_repeat, cluster = fSubject)
## 
##   n= 81505, number of events= 30189 
## 
##                                                  coef exp(coef)  se(coef)
## TrialNo                                      0.014659  1.014767  0.001653
## fCondition25:strata(time_group)time_group=1 -0.001617  0.998384  0.027222
## fCondition50:strata(time_group)time_group=1 -0.083464  0.919924  0.027613
## fCondition75:strata(time_group)time_group=1        NA        NA  0.000000
## fCondition25:strata(time_group)time_group=2 -0.017134  0.983012  0.026689
## fCondition50:strata(time_group)time_group=2  0.036659  1.037339  0.026195
## fCondition75:strata(time_group)time_group=2        NA        NA  0.000000
## fCondition25:strata(time_group)time_group=3 -0.024720  0.975583  0.021370
## fCondition50:strata(time_group)time_group=3  0.178781  1.195758  0.020813
## fCondition75:strata(time_group)time_group=3        NA        NA  0.000000
##                                             robust se      z Pr(>|z|)    
## TrialNo                                      0.001656  8.852  < 2e-16 ***
## fCondition25:strata(time_group)time_group=1  0.025924 -0.062  0.95025    
## fCondition50:strata(time_group)time_group=1  0.025806 -3.234  0.00122 ** 
## fCondition75:strata(time_group)time_group=1  0.000000     NA       NA    
## fCondition25:strata(time_group)time_group=2  0.026494 -0.647  0.51782    
## fCondition50:strata(time_group)time_group=2  0.025512  1.437  0.15074    
## fCondition75:strata(time_group)time_group=2  0.000000     NA       NA    
## fCondition25:strata(time_group)time_group=3  0.020722 -1.193  0.23289    
## fCondition50:strata(time_group)time_group=3  0.020007  8.936  < 2e-16 ***
## fCondition75:strata(time_group)time_group=3  0.000000     NA       NA    
## ---
## Signif. codes:  0 '***' 0.001 '**' 0.01 '*' 0.05 '.' 0.1 ' ' 1
## 
##                                             exp(coef) exp(-coef) lower .95
## TrialNo                                        1.0148     0.9854    1.0115
## fCondition25:strata(time_group)time_group=1    0.9984     1.0016    0.9489
## fCondition50:strata(time_group)time_group=1    0.9199     1.0870    0.8746
## fCondition75:strata(time_group)time_group=1        NA         NA        NA
## fCondition25:strata(time_group)time_group=2    0.9830     1.0173    0.9333
## fCondition50:strata(time_group)time_group=2    1.0373     0.9640    0.9867
## fCondition75:strata(time_group)time_group=2        NA         NA        NA
## fCondition25:strata(time_group)time_group=3    0.9756     1.0250    0.9368
## fCondition50:strata(time_group)time_group=3    1.1958     0.8363    1.1498
## fCondition75:strata(time_group)time_group=3        NA         NA        NA
##                                             upper .95
## TrialNo                                        1.0181
## fCondition25:strata(time_group)time_group=1    1.0504
## fCondition50:strata(time_group)time_group=1    0.9676
## fCondition75:strata(time_group)time_group=1        NA
## fCondition25:strata(time_group)time_group=2    1.0354
## fCondition50:strata(time_group)time_group=2    1.0905
## fCondition75:strata(time_group)time_group=2        NA
## fCondition25:strata(time_group)time_group=3    1.0160
## fCondition50:strata(time_group)time_group=3    1.2436
## fCondition75:strata(time_group)time_group=3        NA
## 
## Concordance= 0.521  (se = 0.002 )
## Likelihood ratio test= 207.3  on 7 df,   p=<2e-16
## Wald test            = 214.4  on 7 df,   p=<2e-16
## Score (logrank) test = 209.7  on 7 df,   p=<2e-16,   Robust = 199.9  p=<2e-16
## 
##   (Note: the likelihood ratio and score tests assume independence of
##      observations within a cluster, the Wald and robust score tests do not).
```

```
AIC(fit_split_repeat)
```

```
## [1] 591587.7
```

```
cox.zph(fit_split_repeat)
```

```
##                                  chisq df    p
## TrialNo                        0.00712  1 0.93
## fCondition:strata(time_group) 10.56939  6 0.10
## GLOBAL                        10.57585  7 0.16
```

```
ggcoxdiagnostics(fit_split_repeat,type="schoenfeld")
```

```
# Now relevel to extract all hazard ratios from the model

# Relevel so condition 50 is the reference level
fit_split_data_repeat$fCondition <- relevel(fit_split_data_repeat$fCondition, "75")
fit_split_repeat <- coxph(Surv(tstart, Time, Hit) ~ fCondition:strata(time_group) + TrialNo, cluster = fSubject, data = fit_split_data_repeat)
summary(fit_split_repeat)

# Relevel so condition 25 is the reference level
fit_split_data_repeat$fCondition <- relevel(fit_split_data_repeat$fCondition, "50")
fit_split_repeat <- coxph(Surv(tstart, Time, Hit) ~ fCondition:strata(time_group) + TrialNo, cluster = fSubject, data = fit_split_data_repeat)
summary(fit_split_repeat)
```

```
# condition only
fit_split_repeat_condonly <- coxph(Surv(tstart, Time, Hit) ~ fCondition:strata(time_group), cluster = fSubject, data = fit_split_data_repeat)
AIC(fit_split_repeat_condonly)
```

```
## [1] 591664.4
```

```
# trial only
fit_split_repeat_trialonly <- coxph(Surv(tstart, Time, Hit) ~ TrialNo, cluster = fSubject, data = fit_split_data_repeat)
AIC(fit_split_repeat_trialonly)
```

```
## [1] 591705.3
```

## Supplementary analyses - repeat main analyses, with only unique plays

```
survival_data_repeat3 <- survival_data %>%
  filter(PlayedBefore == "n")


assumption_fit_unique <- coxph(Surv(Time, Hit) ~ fCondition + TrialNo, cluster = fSubject, data = survival_data_repeat3)
cox.zph(assumption_fit_unique) # assumptions broken
```

```
##            chisq df       p
## fCondition  45.8  2 1.1e-10
## TrialNo     38.9  1 4.6e-10
## GLOBAL      84.4  3 < 2e-16
```

```
# Use survSplit to fit a time-dependent condition variable - with same splits as for full dataset
fit_split_data_unique <- survSplit(Surv(Time, Hit) ~ fCondition + fSubject + TrialNo,
                            data = survival_data_repeat3, cut = c(1.5,2.5), episode = "time_group")

fit_split_unique <- coxph(Surv(tstart, Time, Hit) ~ fCondition:strata(time_group) + TrialNo, cluster = fSubject, data = fit_split_data_unique)
summary(fit_split_unique)
```

```
## Call:
## coxph(formula = Surv(tstart, Time, Hit) ~ fCondition:strata(time_group) + 
##     TrialNo, data = fit_split_data_unique, cluster = fSubject)
## 
##   n= 50734, number of events= 16344 
## 
##                                                  coef exp(coef)  se(coef)
## TrialNo                                      0.031159  1.031649  0.002221
## fCondition25:strata(time_group)time_group=1  0.014366  1.014470  0.043449
## fCondition50:strata(time_group)time_group=1 -0.085524  0.918031  0.044419
## fCondition75:strata(time_group)time_group=1        NA        NA  0.000000
## fCondition25:strata(time_group)time_group=2  0.002096  1.002098  0.035479
## fCondition50:strata(time_group)time_group=2  0.063147  1.065183  0.034902
## fCondition75:strata(time_group)time_group=2        NA        NA  0.000000
## fCondition25:strata(time_group)time_group=3 -0.040729  0.960089  0.027281
## fCondition50:strata(time_group)time_group=3  0.187233  1.205908  0.026493
## fCondition75:strata(time_group)time_group=3        NA        NA  0.000000
##                                             robust se      z Pr(>|z|)    
## TrialNo                                      0.002215 14.068  < 2e-16 ***
## fCondition25:strata(time_group)time_group=1  0.041292  0.348   0.7279    
## fCondition50:strata(time_group)time_group=1  0.042091 -2.032   0.0422 *  
## fCondition75:strata(time_group)time_group=1  0.000000     NA       NA    
## fCondition25:strata(time_group)time_group=2  0.035425  0.059   0.9528    
## fCondition50:strata(time_group)time_group=2  0.034016  1.856   0.0634 .  
## fCondition75:strata(time_group)time_group=2  0.000000     NA       NA    
## fCondition25:strata(time_group)time_group=3  0.026333 -1.547   0.1219    
## fCondition50:strata(time_group)time_group=3  0.026572  7.046 1.84e-12 ***
## fCondition75:strata(time_group)time_group=3  0.000000     NA       NA    
## ---
## Signif. codes:  0 '***' 0.001 '**' 0.01 '*' 0.05 '.' 0.1 ' ' 1
## 
##                                             exp(coef) exp(-coef) lower .95
## TrialNo                                        1.0316     0.9693    1.0272
## fCondition25:strata(time_group)time_group=1    1.0145     0.9857    0.9356
## fCondition50:strata(time_group)time_group=1    0.9180     1.0893    0.8453
## fCondition75:strata(time_group)time_group=1        NA         NA        NA
## fCondition25:strata(time_group)time_group=2    1.0021     0.9979    0.9349
## fCondition50:strata(time_group)time_group=2    1.0652     0.9388    0.9965
## fCondition75:strata(time_group)time_group=2        NA         NA        NA
## fCondition25:strata(time_group)time_group=3    0.9601     1.0416    0.9118
## fCondition50:strata(time_group)time_group=3    1.2059     0.8293    1.1447
## fCondition75:strata(time_group)time_group=3        NA         NA        NA
##                                             upper .95
## TrialNo                                         1.036
## fCondition25:strata(time_group)time_group=1     1.100
## fCondition50:strata(time_group)time_group=1     0.997
## fCondition75:strata(time_group)time_group=1        NA
## fCondition25:strata(time_group)time_group=2     1.074
## fCondition50:strata(time_group)time_group=2     1.139
## fCondition75:strata(time_group)time_group=2        NA
## fCondition25:strata(time_group)time_group=3     1.011
## fCondition50:strata(time_group)time_group=3     1.270
## fCondition75:strata(time_group)time_group=3        NA
## 
## Concordance= 0.542  (se = 0.002 )
## Likelihood ratio test= 288.7  on 7 df,   p=<2e-16
## Wald test            = 285.6  on 7 df,   p=<2e-16
## Score (logrank) test = 290.8  on 7 df,   p=<2e-16,   Robust = 244.2  p=<2e-16
## 
##   (Note: the likelihood ratio and score tests assume independence of
##      observations within a cluster, the Wald and robust score tests do not).
```

```
AIC(fit_split_unique)
```

```
## [1] 305162.9
```

```
cox.zph(fit_split_unique)
```

```
##                               chisq df       p
## TrialNo                        38.6  1 5.2e-10
## fCondition:strata(time_group)  10.8  6   0.096
## GLOBAL                         49.4  7 1.9e-08
```

```
ggcoxdiagnostics(fit_split_unique,type="schoenfeld")
```

```
## `geom_smooth()` using formula 'y ~ x'
```

```
# condition only
fit_split_unique_condonly <- coxph(Surv(tstart, Time, Hit) ~ fCondition:strata(time_group), cluster = fSubject, data = fit_split_data_unique)
AIC(fit_split_unique_condonly)
```

```
## [1] 305357.8
```

```
# trial only
fit_split_unique_trialonly <- coxph(Surv(tstart, Time, Hit) ~ TrialNo, cluster = fSubject, data = fit_split_data_unique)
AIC(fit_split_unique_trialonly)
```

```
## [1] 305244.2
```

## EXPERIMENT 2

### Load and prepare data

```
DATA2 <- read_csv('full_data_Expt2.csv')#for survival analyses
```

BackgroundType variable defines one of four types: Bark, Leaves, Grass, Shrub.

In the moth\_background\_letters column: specialists are A,B,C,D - A is bark, B is grass, C is leaves, D is shrub. So if type is bark and moth is A -> target is a matched specialist; if moths are B, C, or D -> target is mismatched. And so on for other background types.

```
DATA2$Matched<-DATA2$Condition
for (i in 1:length(DATA2$TrialNo)){
  if(DATA2$moth_background_letters[i]=="A"){
    if(DATA2$BackgroundType[i]=="Bark"){
      DATA2$Matched[i]<-"S_matched"
    }
    else {
      DATA2$Matched[i]<-"S_mismatched"
    }
  }
  else if (DATA2$moth_background_letters[i]=="B"){
    if(DATA2$BackgroundType[i]=="Grass"){
      DATA2$Matched[i]<-"S_matched"
    }
    else {
      DATA2$Matched[i]<-"S_mismatched"
    }
  }
  else if (DATA2$moth_background_letters[i]=="C"){
    if(DATA2$BackgroundType[i]=="Leaves"){
      DATA2$Matched[i]<-"S_matched"
    }
    else {
      DATA2$Matched[i]<-"S_mismatched"
    }
  }
  else if (DATA2$moth_background_letters[i]=="D"){
    if(DATA2$BackgroundType[i]=="Shrub"){
      DATA2$Matched[i]<-"S_matched"
    }
    else {
      DATA2$Matched[i]<-"S_mismatched"
    }
  }
}
```

To determine how many volunteers have taken part in the experiment:

```
#How many players in total?
num_players <- DATA2 %>%
  group_by(Subject) %>%
  summarise(total = n_distinct(ParticipantNum))  

#How many players without mobiles?
DATA2_MOBEX<-DATA2%>%
  filter(Device!="mobile")
num_players_ex <- DATA2_MOBEX %>%
  group_by(Subject) %>%
  summarise(total = n_distinct(ParticipantNum)) 

#And how many unique players? - players who haven't played before
num_players_unique <- DATA2 %>%
  filter(PlayedBefore=="n")%>%
  group_by(Subject) %>%
  summarise(total = n_distinct(ParticipantNum)) 

num_players_unique_mobex <- DATA2_MOBEX %>%
  filter(PlayedBefore=="n")%>%
  group_by(Subject) %>%
  summarise(total = n_distinct(ParticipantNum)) 

#analyses will focus on all players excluding mobile devices
```

Each “folder”(set of moths) was played 4 - 20 times.  
1228 plays in total, with 940 unique players.  
Excluding plays on mobiles, we have 1204 plays, with 921 unique players.

To determine how many incorrect clicks occurred before participants found the target:

```
ClickSummary2<-DATA2_MOBEX%>%
  group_by(TrialNo, ParticipantNum)%>%
  summarise (click_number=n())#this data frame shows how many times each participant clicked for each trial
```

```
## `summarise()` has grouped output by 'TrialNo'. You can override using the
## `.groups` argument.
```

```
#How many trials with more than 5 clicks?
ManyClicks2<-ClickSummary2%>%
  filter(click_number>5)
length(ManyClicks2$ParticipantNum)#2229 trials, out of 43344 in total
length(unique(ManyClicks2$ParticipantNum))#involves 636 participants

PercentLargeClicks2<-100*length(ManyClicks2$TrialNo)/length(ClickSummary2$TrialNo)#only 5.14% trials have > 5 clicks
```

The median number of clicks per target is 1 (mean = 1.7274363), so in most cases, participants only click when they spot the target. However, a small number of trials include larger numbers of clicks: the maximum number of clicks for a single trial is 51, but only 5.1425803 % of trials have over 5 clicks.

```
# Remove all but final clicks for each slide 
SURVDATA2_MOBEX <- DATA2_MOBEX %>%
  group_by(ParticipantNum,TrialNo) %>%
  slice(n()) %>%
  ungroup()
#str(SURVDATA2_MOBEX)#43344 rows - 1204 plays with 36 trials each

# Detection time -  replace NAs with timeout time (15s)
SURVDATA2_MOBEX$Time <- SURVDATA2_MOBEX$Time %>% replace_na(15000)

# Divide time by 1000 to convert detection time to s, rather than ms
SURVDATA2_MOBEX$Time <- SURVDATA2_MOBEX$Time/1000

# Add start column (0) for model syntax
SURVDATA2_MOBEX$Start<-rep(0,length(SURVDATA2_MOBEX$Subject))
```

### Odds ratios

Data for odds ratio calculations:

```
survival_data_OR_Expt2 <- SURVDATA2_MOBEX %>%
  group_by(Condition) %>%
  summarise(Total = n(), Total_hits = sum(Hit))
```

Number of targets and hits per condition in Experiment 2

| Condition | Total | Total\_hits |
| --- | --- | --- |
| G1 | 14448 | 11978 |
| G2 | 14448 | 12092 |
| S | 14448 | 12006 |

```
# odds ratio relative to S
condition <- c("S", "G1", "G2")
hit <- c("Yes", "No")
dat <- matrix(c(12006, 2442, 11978, 2470, 12092, 2356), nrow = 3, ncol = 2, byrow = TRUE)
dimnames(dat) <- list("Condition" = condition, "Hit" = hit)
or_expt2_S<-oddsratio(dat)

# odds ratio relative to G1
condition <- c("G1", "G2","S")
hit <- c("Yes", "No")
dat <- matrix(c(11978, 2470, 12092, 2356,12006, 2442), nrow = 3, ncol = 2, byrow = TRUE)
dimnames(dat) <- list("Condition" = condition, "Hit" = hit)
or_expt2_G1<-oddsratio(dat)
```

Results from odds ratio calculations:  

Odds ratios relative to S

|  | estimate | lower | upper |
| --- | --- | --- | --- |
| S | 1.000 | NA | NA |
| G1 | 1.014 | 0.953 | 1.078 |
| G2 | 0.958 | 0.900 | 1.019 |

Statistical tests for odds ratios (relative to S)

|  | midp.exact | fisher.exact | chi.square |
| --- | --- | --- | --- |
| S | NA | NA | NA |
| G1 | 0.661 | 0.672 | 0.661 |
| G2 | 0.174 | 0.179 | 0.174 |

Odds ratios relative to G1

|  | estimate | lower | upper |
| --- | --- | --- | --- |
| G1 | 1.000 | NA | NA |
| G2 | 0.945 | 0.888 | 1.005 |
| S | 0.986 | 0.928 | 1.049 |

Statistical tests for odds ratios (relative to G1)

|  | midp.exact | fisher.exact | chi.square |
| --- | --- | --- | --- |
| G1 | NA | NA | NA |
| G2 | 0.072 | 0.075 | 0.072 |
| S | 0.661 | 0.672 | 0.661 |

### Plot survival curves for Experiment 2

```
SURVDATA2_MOBEX$Condition<-as.factor(SURVDATA2_MOBEX$Condition)
SURVDATA2_MOBEX$Condition<-relevel(SURVDATA2_MOBEX$Condition, ref="S")#relevel so S is reference
fit <- survfit(Surv(Time, Hit) ~ Condition, data = SURVDATA2_MOBEX, cluster = ParticipantNum, type = "kaplan-meier")
# main plot
p<-ggsurvplot(fit, data = SURVDATA2_MOBEX, conf.int = TRUE, pval = FALSE, break.time.by = 1,  
              palette = c("#E69F00", "#56B4E9","#0072B2"),
              legend.title = "Condition:", 
              legend.labs=c("S","G1","G2"),
              font.x = 24, font.y = 24, 
              font.tickslab = 18, font.legend = 18, xlab = "Time (s)")
# zoomed in inset:
zoom <- ggsurvplot(fit, data = SURVDATA2_MOBEX, conf.int = TRUE, risk.table = FALSE, break.time.by = 0.5,
                   xlim = c(0,2),ylim = c(0.55,1),
                   palette = c("#E69F00", "#56B4E9","#0072B2"),
                   legend="none",
                   font.x = 18, font.y = 18, 
                   font.tickslab = 16, font.legend = 18, xlab = "Time (s)")

# plot with both full survival curve and zoomed in inset
p2 <- p$plot + 
  annotation_custom(grob = ggplotGrob(zoom$plot),
                    xmin = 7,xmax=Inf,ymin = .34,ymax = 1)
p2
```

```
# to save for paper
ggsave("Fig5.tiff", device='tiff', dpi=300)
```

**Figure 5** Survival probability for specialist (S) and generalist (G1,G2) targets in Experiment 2.

### Survival analyses

As in experiment 1, initial models violated the proportional hazards assumption, so target condition was fitted as a time-varying coefficient, improving the diagnostics. Trial number, condition and distance from the centre of the screen were included as fixed effects, and participant number was added as a cluster term.

```
#initial model - assumption is violated
phmodel1<-coxph(Surv(Start,Time,Hit)~Condition+TrialNo+DistFromCentre, cluster = ParticipantNum, 
                data=SURVDATA2_MOBEX) 
summary(phmodel1)
```

```
cox.zph(phmodel1)
```

```
##                chisq df       p
## Condition        126  2 < 2e-16
## TrialNo           20  1 7.9e-06
## DistFromCentre  1720  1 < 2e-16
## GLOBAL          1873  4 < 2e-16
```

```
#ggcoxdiagnostics(phmodel1, type="schoenfeld") #deviation in line, at start and end
```

```
# Time-dependent model
  #time splits based on inspecting the survival plot: lines cross approximately at 0.6, 1.2, 1.4, 2, and 10.5 s

# split data
SURVDATA2_MOBEX_SPLIT<-survSplit(Surv(Time, Hit) ~ Condition+TrialNo+ParticipantNum+DistFromCentre, data=SURVDATA2_MOBEX, cut=c(0.6, 1.2, 1.4, 2,10.5), episode="tgroup", id="id")

# run time-dependent model
tdmodel1<-coxph(Surv(tstart,Time,Hit)~Condition:strata(tgroup)+TrialNo+DistFromCentre, cluster = ParticipantNum,data=SURVDATA2_MOBEX_SPLIT) 
summary(tdmodel1)
```

```
ggcoxdiagnostics(tdmodel1, type="schoenfeld") #straighter line for diagnostics - and better than other time splits
```

The biggest deviation in the diagnostic plots is linked to the “distance from centre” variable, so the next model stratifies the data by distance from centre instead.

```
#prepare data by splitting distance from centre into quartiles
summary(SURVDATA2_MOBEX$DistFromCentre)
SURVDATA2_MOBEX$DistFromCentreQuart<-rep(0,length(SURVDATA2_MOBEX$Subject))
for (i in 1:length(SURVDATA2_MOBEX$Subject)){
  if(SURVDATA2_MOBEX$DistFromCentre[i]<=346.358){
    SURVDATA2_MOBEX$DistFromCentreQuart[i]<-1
  }
  else if(SURVDATA2_MOBEX$DistFromCentre[i]>346.358 && SURVDATA2_MOBEX$DistFromCentre[i]<=491.648){
    SURVDATA2_MOBEX$DistFromCentreQuart[i]<-2
  }
  else if(SURVDATA2_MOBEX$DistFromCentre[i]>491.648 && SURVDATA2_MOBEX$DistFromCentre[i]<=665.018){
    SURVDATA2_MOBEX$DistFromCentreQuart[i]<-3
  }
  else{SURVDATA2_MOBEX$DistFromCentreQuart[i]<-4}
}

# best split, following plot, and with low deviations for prop hazards plots (though cox.zph p values break assumption)
SURVDATA2_MOBEX_SPLIT<-survSplit(Surv(Time, Hit) ~ Condition+TrialNo+ParticipantNum+DistFromCentreQuart, data=SURVDATA2_MOBEX, cut=c(0.6, 1.2,1.4,2,10.5), episode="tgroup", id="id")

tdmodel2<-coxph(Surv(tstart,Time,Hit)~Condition:strata(tgroup)+TrialNo+strata(DistFromCentreQuart), cluster = ParticipantNum,data=SURVDATA2_MOBEX_SPLIT)
```

```
cox.zph(tdmodel2)
```

```
##                          chisq df       p
## TrialNo                   19.3  1 1.1e-05
## Condition:strata(tgroup)  68.5 12 6.2e-10
## GLOBAL                    87.3 13 4.6e-13
```

```
ggcoxdiagnostics(tdmodel2, type="schoenfeld") #straighter line for diagnostics - ok
```

Though the assumption is still broken according to strict p-value tests, diagnostic plots suggest deviations are very small, so this model is appropriate.

Next, we test the significance of each fixed effect, and relevel the model by condition to extract hazard ratios.

```
# test effects of trial number and condition:time
tdmodel2b<-coxph(Surv(tstart,Time,Hit)~Condition:strata(tgroup)+strata(DistFromCentreQuart), cluster = ParticipantNum,data=SURVDATA2_MOBEX_SPLIT)
tdmodel2c<-coxph(Surv(tstart,Time,Hit)~TrialNo+strata(DistFromCentreQuart), cluster = ParticipantNum,data=SURVDATA2_MOBEX_SPLIT)


# relevel to get HRs relative to each condition

SURVDATA2_MOBEX_SPLIT$Condition<-factor(SURVDATA2_MOBEX_SPLIT$Condition, levels=c("S","G1","G2"))
tdmodel2<-coxph(Surv(tstart,Time,Hit)~Condition:strata(tgroup)+TrialNo+strata(DistFromCentreQuart), cluster = ParticipantNum,data=SURVDATA2_MOBEX_SPLIT) 
summary(tdmodel2)

SURVDATA2_MOBEX_SPLIT$Condition<-factor(SURVDATA2_MOBEX_SPLIT$Condition, levels=c("G1","G2","S"))
tdmodel2<-coxph(Surv(tstart,Time,Hit)~Condition:strata(tgroup)+TrialNo+strata(DistFromCentreQuart), cluster= ParticipantNum,data=SURVDATA2_MOBEX_SPLIT) 
summary(tdmodel2)

SURVDATA2_MOBEX_SPLIT$Condition<-factor(SURVDATA2_MOBEX_SPLIT$Condition, levels=c("S","G2","G1"))
tdmodel2<-coxph(Surv(tstart,Time,Hit)~Condition:strata(tgroup)+TrialNo+strata(DistFromCentreQuart), cluster = ParticipantNum,data=SURVDATA2_MOBEX_SPLIT) 
summary(tdmodel2)
```

Both trial number and time-dependent condition have a significant effect on survival:

```
# full model
AIC(tdmodel2)
```

```
## [1] 623914.7
```

```
# effect of trial number
AIC(tdmodel2b)
```

```
## [1] 624037.1
```

```
# effect of condition:time
AIC(tdmodel2c)
```

```
## [1] 624017.5
```

### Supplementary analyses - plots splitting specialists into matched/mismatched types

Additional analyses, separating out specialist targets that are made from the same type of background they are shown on, or not.

```
# Setting up for graph 
SURVDATA2_MOBEX$Matched<-as.factor(SURVDATA2_MOBEX$Matched)
SURVDATA2_MOBEX$Matched<-factor(SURVDATA2_MOBEX$Matched, levels=c("G1","G2","S_matched","S_mismatched"))
graph_fit2 <- survfit(Surv(Time, Hit) ~ Matched, data = SURVDATA2_MOBEX, cluster = ParticipantNum, type = "kaplan-meier")

# Plotting survival curves 
main_plot2 <- ggsurvplot(graph_fit2, data = SURVDATA2_MOBEX, conf.int = TRUE, 
                        pval = FALSE, break.time.by = 1,
                        legend.title = "Condition :",
                        legend.labs = c( "Generalist (G1)","Generalist (G2)", "Matched Specialist","Mismatched Specialist"), 
                        palette = c("#56B4E9","#0072B2","#E69F00", "#009E73"), 
                        font.x = 24, font.y = 24, 
                        font.tickslab = 20, font.legend = 12,
                        xlab = "Time (s)")
main_plot2
```

**Supplementary Figure 6** Survival probability for specialist (matched/mismatched) and generalist (G1/G2) targets in Experiment 2.

We can also plot time of capture for matched/mismatched specialists and generalists:

```
ggplot(SURVDATA2_MOBEX, aes(x=Time, fill=Matched)) + 
  geom_histogram(aes(y = stat(density)), position="dodge")+
  scale_y_continuous(labels = scales::percent_format(suffix="",accuracy=1), 
                     limits = c(0,0.9), expand = c(0, 0))+
   scale_x_continuous(expand = c(0, 0))+
   scale_fill_manual(name="Condition", values=c("#56B4E9","#0072B2","#E69F00", "#009E73"), labels=c("G1","G2", "Matched Specialist","Mismatched Specialist"))+
   xlab("Time (s)") + ylab("Targets captured (%)")+
   my_theme+ theme(legend.position="top")
```

**Supplementary Figure 7** Histogram of capture times for specialist (matched/mismatched) and generalist (G1/G2) targets in Experiment 2.

### Supplementary analyses - repeat main analyses, excluding plays with over 5 clicks

```
#Step 1: remove plays where participants made > 5 clicks
ManyClicks2$Subj_Trial<-paste(ManyClicks2$ParticipantNum,ManyClicks2$TrialNo,sep="_")
survival_data_repeat_exp2<-SURVDATA2_MOBEX
survival_data_repeat_exp2$TooManyClicks<-rep("no",length(survival_data_repeat_exp2$TrialNo))
survival_data_repeat_exp2$Subj_Trial<-paste(survival_data_repeat_exp2$ParticipantNum,
                                       survival_data_repeat_exp2$TrialNo,sep="_")

for (i in 1:length(survival_data_repeat_exp2$TrialNo)){
  for(j in 1:length(ManyClicks2$Subj_Trial)){
    if(survival_data_repeat_exp2$Subj_Trial[i]==ManyClicks2$Subj_Trial[j]){
      survival_data_repeat_exp2$TooManyClicks[i]<-"yes"
    }
  }
  #print(i)
}

library(tidyverse)
survival_data_repeat_exp2_2<-survival_data_repeat_exp2%>%
  filter(TooManyClicks!="yes")#43344-2229=41115 rows now

#Step 2: repeat survival analyses

#Basic survival model - assumptions not met

phmodel1_repeat<-coxph(Surv(Start,Time,Hit)~Condition+TrialNo+DistFromCentre, cluster = ParticipantNum,data=survival_data_repeat_exp2_2) 
summary(phmodel1_repeat)
```

```
cox.zph(phmodel1_repeat) #assumptions not met for any variable
```

```
##                 chisq df      p
## Condition       125.0  2 <2e-16
## TrialNo          20.5  1  6e-06
## DistFromCentre 1748.9  1 <2e-16
## GLOBAL         1907.8  4 <2e-16
```

```
# Time-dependent model - time splits as for full dataset

# split data
SURVDATA2_MOBEX_SPLIT_repeat<-survSplit(Surv(Time, Hit) ~ Condition+TrialNo+ParticipantNum+DistFromCentre, data=survival_data_repeat_exp2_2, cut=c(0.6, 1.2, 1.4, 2,10.5), episode="tgroup", id="id")

# run time-dependent model
tdmodel1_repeat<-coxph(Surv(tstart,Time,Hit)~Condition:strata(tgroup)+TrialNo+DistFromCentre, cluster = ParticipantNum,data=SURVDATA2_MOBEX_SPLIT_repeat) 
summary(tdmodel1_repeat)

#ggcoxdiagnostics(tdmodel1_repeat, type="schoenfeld") #straighter line for diagnostics - and better than other time splits

#As for original analysis, stratify the data by distance from centre instead.
#prepare data by splitting distance from centre into quartiles
summary(survival_data_repeat_exp2_2$DistFromCentre)#new values for changed datatset
survival_data_repeat_exp2_2$DistFromCentreQuart<-rep(0,length(survival_data_repeat_exp2_2$Subject))
for (i in 1:length(survival_data_repeat_exp2_2$Subject)){
  if(survival_data_repeat_exp2_2$DistFromCentre[i]<=345.146){
    survival_data_repeat_exp2_2$DistFromCentreQuart[i]<-1
  }
  else if(survival_data_repeat_exp2_2$DistFromCentre[i]>345.146 && survival_data_repeat_exp2_2$DistFromCentre[i]<=490.898){
    survival_data_repeat_exp2_2$DistFromCentreQuart[i]<-2
  }
  else if(survival_data_repeat_exp2_2$DistFromCentre[i]>490.898 && survival_data_repeat_exp2_2$DistFromCentre[i]<=664.313){
    survival_data_repeat_exp2_2$DistFromCentreQuart[i]<-3
  }
  else{survival_data_repeat_exp2_2$DistFromCentreQuart[i]<-4}
}

# best split, following plot, and with low deviations for prop hazards plots (though cox.zph p values still break assumption)
SURVDATA2_MOBEX_SPLIT_repeat<-survSplit(Surv(Time, Hit) ~ Condition+TrialNo+ParticipantNum+DistFromCentreQuart, data=survival_data_repeat_exp2_2, cut=c(0.6, 1.2,1.4,2,10.5), episode="tgroup", id="id")

tdmodel2_repeat<-coxph(Surv(tstart,Time,Hit)~Condition:strata(tgroup)+TrialNo+strata(DistFromCentreQuart), cluster = ParticipantNum,data=SURVDATA2_MOBEX_SPLIT_repeat)
```

```
#cox.zph(tdmodel2_repeat)
ggcoxdiagnostics(tdmodel2_repeat, type="schoenfeld") #straighter line for diagnostics - ok
```

```
#Next, we test the significance of each fixed effect, and relevel the model by condition to extract hazard ratios.  

# test effects of trial number and condition:time - effects as for full dataset
tdmodel2b_repeat<-coxph(Surv(tstart,Time,Hit)~Condition:strata(tgroup)+strata(DistFromCentreQuart), cluster = ParticipantNum,data=SURVDATA2_MOBEX_SPLIT_repeat)
tdmodel2c_repeat<-coxph(Surv(tstart,Time,Hit)~TrialNo+strata(DistFromCentreQuart), cluster =  ParticipantNum,data=SURVDATA2_MOBEX_SPLIT_repeat)
```

Both trial number and time-dependent condition have a significant effect on survival:

```
# full model
AIC(tdmodel2_repeat)
```

```
## [1] 605798.7
```

```
# effect of trial
AIC(tdmodel2b_repeat)
```

```
## [1] 605880.5
```

```
# effect of condition
AIC(tdmodel2c_repeat)
```

```
## [1] 605900.5
```

```
# Relevel to get HRs relative to each condition
summary(tdmodel2_repeat)
```

```
## Call:
## coxph(formula = Surv(tstart, Time, Hit) ~ Condition:strata(tgroup) + 
##     TrialNo + strata(DistFromCentreQuart), data = SURVDATA2_MOBEX_SPLIT_repeat, 
##     cluster = ParticipantNum)
## 
##   n= 197107, number of events= 35413 
## 
##                                          coef  exp(coef)   se(coef)  robust se
## TrialNo                             0.0046487  1.0046595  0.0005078  0.0006044
## ConditionS:strata(tgroup)tgroup=1   0.2012905  1.2229800  0.3733957  0.2976413
## ConditionG1:strata(tgroup)tgroup=1 -0.7868611  0.4552716  0.4935527  0.5340201
## ConditionG2:strata(tgroup)tgroup=1         NA         NA  0.0000000  0.0000000
## ConditionS:strata(tgroup)tgroup=2   0.2743601  1.3156885  0.0555059  0.0519922
## ConditionG1:strata(tgroup)tgroup=2  0.0370814  1.0377775  0.0584820  0.0551426
## ConditionG2:strata(tgroup)tgroup=2         NA         NA  0.0000000  0.0000000
## ConditionS:strata(tgroup)tgroup=3   0.2245868  1.2518054  0.0567944  0.0553212
## ConditionG1:strata(tgroup)tgroup=3 -0.0413906  0.9594543  0.0602061  0.0622403
## ConditionG2:strata(tgroup)tgroup=3         NA         NA  0.0000000  0.0000000
## ConditionS:strata(tgroup)tgroup=4   0.1694208  1.1846185  0.0296993  0.0276054
## ConditionG1:strata(tgroup)tgroup=4  0.0018101  1.0018118  0.0306335  0.0295488
## ConditionG2:strata(tgroup)tgroup=4         NA         NA  0.0000000  0.0000000
## ConditionS:strata(tgroup)tgroup=5  -0.0089915  0.9910488  0.0162032  0.0157149
## ConditionG1:strata(tgroup)tgroup=5 -0.0399481  0.9608393  0.0159852  0.0145442
## ConditionG2:strata(tgroup)tgroup=5         NA         NA  0.0000000  0.0000000
## ConditionS:strata(tgroup)tgroup=6  -0.1637508  0.8489536  0.0565902  0.0565301
## ConditionG1:strata(tgroup)tgroup=6  0.0473482  1.0484870  0.0534331  0.0521404
## ConditionG2:strata(tgroup)tgroup=6         NA         NA  0.0000000  0.0000000
##                                         z Pr(>|z|)    
## TrialNo                             7.692 1.45e-14 ***
## ConditionS:strata(tgroup)tgroup=1   0.676  0.49886    
## ConditionG1:strata(tgroup)tgroup=1 -1.473  0.14063    
## ConditionG2:strata(tgroup)tgroup=1     NA       NA    
## ConditionS:strata(tgroup)tgroup=2   5.277 1.31e-07 ***
## ConditionG1:strata(tgroup)tgroup=2  0.672  0.50129    
## ConditionG2:strata(tgroup)tgroup=2     NA       NA    
## ConditionS:strata(tgroup)tgroup=3   4.060 4.91e-05 ***
## ConditionG1:strata(tgroup)tgroup=3 -0.665  0.50604    
## ConditionG2:strata(tgroup)tgroup=3     NA       NA    
## ConditionS:strata(tgroup)tgroup=4   6.137 8.40e-10 ***
## ConditionG1:strata(tgroup)tgroup=4  0.061  0.95115    
## ConditionG2:strata(tgroup)tgroup=4     NA       NA    
## ConditionS:strata(tgroup)tgroup=5  -0.572  0.56721    
## ConditionG1:strata(tgroup)tgroup=5 -2.747  0.00602 ** 
## ConditionG2:strata(tgroup)tgroup=5     NA       NA    
## ConditionS:strata(tgroup)tgroup=6  -2.897  0.00377 ** 
## ConditionG1:strata(tgroup)tgroup=6  0.908  0.36383    
## ConditionG2:strata(tgroup)tgroup=6     NA       NA    
## ---
## Signif. codes:  0 '***' 0.001 '**' 0.01 '*' 0.05 '.' 0.1 ' ' 1
## 
##                                    exp(coef) exp(-coef) lower .95 upper .95
## TrialNo                               1.0047     0.9954    1.0035    1.0059
## ConditionS:strata(tgroup)tgroup=1     1.2230     0.8177    0.6824    2.1917
## ConditionG1:strata(tgroup)tgroup=1    0.4553     2.1965    0.1598    1.2967
## ConditionG2:strata(tgroup)tgroup=1        NA         NA        NA        NA
## ConditionS:strata(tgroup)tgroup=2     1.3157     0.7601    1.1882    1.4568
## ConditionG1:strata(tgroup)tgroup=2    1.0378     0.9636    0.9315    1.1562
## ConditionG2:strata(tgroup)tgroup=2        NA         NA        NA        NA
## ConditionS:strata(tgroup)tgroup=3     1.2518     0.7988    1.1232    1.3952
## ConditionG1:strata(tgroup)tgroup=3    0.9595     1.0423    0.8493    1.0839
## ConditionG2:strata(tgroup)tgroup=3        NA         NA        NA        NA
## ConditionS:strata(tgroup)tgroup=4     1.1846     0.8442    1.1222    1.2505
## ConditionG1:strata(tgroup)tgroup=4    1.0018     0.9982    0.9454    1.0615
## ConditionG2:strata(tgroup)tgroup=4        NA         NA        NA        NA
## ConditionS:strata(tgroup)tgroup=5     0.9910     1.0090    0.9610    1.0220
## ConditionG1:strata(tgroup)tgroup=5    0.9608     1.0408    0.9338    0.9886
## ConditionG2:strata(tgroup)tgroup=5        NA         NA        NA        NA
## ConditionS:strata(tgroup)tgroup=6     0.8490     1.1779    0.7599    0.9484
## ConditionG1:strata(tgroup)tgroup=6    1.0485     0.9538    0.9466    1.1613
## ConditionG2:strata(tgroup)tgroup=6        NA         NA        NA        NA
## 
## Concordance= 0.524  (se = 0.002 )
## Likelihood ratio test= 210.1  on 13 df,   p=<2e-16
## Wald test            = 228.1  on 13 df,   p=<2e-16
## Score (logrank) test = 211.6  on 13 df,   p=<2e-16,   Robust = 187.9  p=<2e-16
## 
##   (Note: the likelihood ratio and score tests assume independence of
##      observations within a cluster, the Wald and robust score tests do not).
```

```
#relative to G1
SURVDATA2_MOBEX_SPLIT_repeat$Condition<-factor(SURVDATA2_MOBEX_SPLIT_repeat$Condition, levels=c("S","G2","G1"))
tdmodel2_repeat<-coxph(Surv(tstart,Time,Hit)~Condition:strata(tgroup)+TrialNo+strata(DistFromCentreQuart), cluster = ParticipantNum,data=SURVDATA2_MOBEX_SPLIT_repeat) 
summary(tdmodel2_repeat)

#relative to S
SURVDATA2_MOBEX_SPLIT_repeat$Condition<-factor(SURVDATA2_MOBEX_SPLIT_repeat$Condition, levels=c("G1","G2","S"))
tdmodel2_repeat<-coxph(Surv(tstart,Time,Hit)~Condition:strata(tgroup)+TrialNo+strata(DistFromCentreQuart), cluster = ParticipantNum,data=SURVDATA2_MOBEX_SPLIT_repeat) 
summary(tdmodel2_repeat)

#relative to G2
SURVDATA2_MOBEX_SPLIT_repeat$Condition<-factor(SURVDATA2_MOBEX_SPLIT_repeat$Condition, levels=c("S","G1","G2"))
tdmodel2_repeat<-coxph(Surv(tstart,Time,Hit)~Condition:strata(tgroup)+TrialNo+strata(DistFromCentreQuart), cluster = ParticipantNum,data=SURVDATA2_MOBEX_SPLIT_repeat) 
summary(tdmodel2_repeat)

#HR tables show that the pattern of HRs is the same as for the full dataset
```

## Supplementary analyses - repeat main analyses, with only unique plays

```
survival_data_repeat_exp2_3<-survival_data_repeat_exp2%>%
  filter(PlayedBefore == "n")

phmodel1_unique<-coxph(Surv(Start,Time,Hit)~Condition+TrialNo+DistFromCentre, cluster = ParticipantNum,data=survival_data_repeat_exp2_3) 
summary(phmodel1_unique)
```

```
## Call:
## coxph(formula = Surv(Start, Time, Hit) ~ Condition + TrialNo + 
##     DistFromCentre, data = survival_data_repeat_exp2_3, cluster = ParticipantNum)
## 
##   n= 33156, number of events= 27196 
## 
##                      coef  exp(coef)   se(coef)  robust se       z Pr(>|z|)    
## ConditionG1    -8.645e-02  9.172e-01  1.486e-02  1.325e-02  -6.524 6.86e-11 ***
## ConditionG2    -7.042e-02  9.320e-01  1.483e-02  1.345e-02  -5.235 1.65e-07 ***
## TrialNo         6.583e-03  1.007e+00  5.740e-04  6.883e-04   9.564  < 2e-16 ***
## DistFromCentre -4.900e-04  9.995e-01  2.973e-05  3.186e-05 -15.379  < 2e-16 ***
## ---
## Signif. codes:  0 '***' 0.001 '**' 0.01 '*' 0.05 '.' 0.1 ' ' 1
## 
##                exp(coef) exp(-coef) lower .95 upper .95
## ConditionG1       0.9172     1.0903    0.8937    0.9413
## ConditionG2       0.9320     1.0730    0.9078    0.9569
## TrialNo           1.0066     0.9934    1.0052    1.0080
## DistFromCentre    0.9995     1.0005    0.9994    0.9996
## 
## Concordance= 0.559  (se = 0.002 )
## Likelihood ratio test= 438.1  on 4 df,   p=<2e-16
## Wald test            = 367.2  on 4 df,   p=<2e-16
## Score (logrank) test = 439.4  on 4 df,   p=<2e-16,   Robust = 263.5  p=<2e-16
## 
##   (Note: the likelihood ratio and score tests assume independence of
##      observations within a cluster, the Wald and robust score tests do not).
```

```
cox.zph(phmodel1_unique)
```

```
##                 chisq df       p
## Condition       100.7  2 < 2e-16
## TrialNo          25.4  1 4.6e-07
## DistFromCentre 1312.3  1 < 2e-16
## GLOBAL         1445.9  4 < 2e-16
```

```
# split data
SURVDATA2_MOBEX_SPLIT_unique<-survSplit(Surv(Time, Hit) ~ Condition+TrialNo+ParticipantNum+DistFromCentre, data=survival_data_repeat_exp2_3, cut=c(0.6, 1.2, 1.4, 2,10.5), episode="tgroup", id="id")

# run time-dependent model
tdmodel1_unique<-coxph(Surv(tstart,Time,Hit)~Condition:strata(tgroup)+TrialNo+DistFromCentre, cluster = ParticipantNum,data=SURVDATA2_MOBEX_SPLIT_unique) 
summary(tdmodel1_unique)
```

```
## Call:
## coxph(formula = Surv(tstart, Time, Hit) ~ Condition:strata(tgroup) + 
##     TrialNo + DistFromCentre, data = SURVDATA2_MOBEX_SPLIT_unique, 
##     cluster = ParticipantNum)
## 
##   n= 163091, number of events= 27196 
## 
##                                          coef  exp(coef)   se(coef)  robust se
## TrialNo                             6.611e-03  1.007e+00  5.740e-04  6.877e-04
## DistFromCentre                     -4.900e-04  9.995e-01  2.973e-05  3.181e-05
## ConditionS:strata(tgroup)tgroup=1  -1.098e+00  3.334e-01  8.165e-01  7.070e-01
## ConditionG1:strata(tgroup)tgroup=1 -1.792e+00  1.666e-01  1.080e+00  1.080e+00
## ConditionG2:strata(tgroup)tgroup=1         NA         NA  0.000e+00  0.000e+00
## ConditionS:strata(tgroup)tgroup=2   3.814e-01  1.464e+00  7.552e-02  6.555e-02
## ConditionG1:strata(tgroup)tgroup=2  9.167e-02  1.096e+00  8.040e-02  7.790e-02
## ConditionG2:strata(tgroup)tgroup=2         NA         NA  0.000e+00  0.000e+00
## ConditionS:strata(tgroup)tgroup=3   2.787e-01  1.321e+00  6.901e-02  6.949e-02
## ConditionG1:strata(tgroup)tgroup=3  1.499e-02  1.015e+00  7.308e-02  7.695e-02
## ConditionG2:strata(tgroup)tgroup=3         NA         NA  0.000e+00  0.000e+00
## ConditionS:strata(tgroup)tgroup=4   2.026e-01  1.225e+00  3.486e-02  3.228e-02
## ConditionG1:strata(tgroup)tgroup=4  5.468e-02  1.056e+00  3.580e-02  3.462e-02
## ConditionG2:strata(tgroup)tgroup=4         NA         NA  0.000e+00  0.000e+00
## ConditionS:strata(tgroup)tgroup=5   2.278e-02  1.023e+00  1.821e-02  1.747e-02
## ConditionG1:strata(tgroup)tgroup=5 -4.662e-02  9.544e-01  1.808e-02  1.643e-02
## ConditionG2:strata(tgroup)tgroup=5         NA         NA  0.000e+00  0.000e+00
## ConditionS:strata(tgroup)tgroup=6  -1.381e-01  8.710e-01  5.773e-02  5.735e-02
## ConditionG1:strata(tgroup)tgroup=6  3.406e-02  1.035e+00  5.443e-02  5.336e-02
## ConditionG2:strata(tgroup)tgroup=6         NA         NA  0.000e+00  0.000e+00
##                                          z Pr(>|z|)    
## TrialNo                              9.614  < 2e-16 ***
## DistFromCentre                     -15.405  < 2e-16 ***
## ConditionS:strata(tgroup)tgroup=1   -1.554  0.12027    
## ConditionG1:strata(tgroup)tgroup=1  -1.659  0.09715 .  
## ConditionG2:strata(tgroup)tgroup=1      NA       NA    
## ConditionS:strata(tgroup)tgroup=2    5.819 5.92e-09 ***
## ConditionG1:strata(tgroup)tgroup=2   1.177  0.23928    
## ConditionG2:strata(tgroup)tgroup=2      NA       NA    
## ConditionS:strata(tgroup)tgroup=3    4.010 6.07e-05 ***
## ConditionG1:strata(tgroup)tgroup=3   0.195  0.84550    
## ConditionG2:strata(tgroup)tgroup=3      NA       NA    
## ConditionS:strata(tgroup)tgroup=4    6.277 3.46e-10 ***
## ConditionG1:strata(tgroup)tgroup=4   1.579  0.11424    
## ConditionG2:strata(tgroup)tgroup=4      NA       NA    
## ConditionS:strata(tgroup)tgroup=5    1.304  0.19210    
## ConditionG1:strata(tgroup)tgroup=5  -2.838  0.00454 ** 
## ConditionG2:strata(tgroup)tgroup=5      NA       NA    
## ConditionS:strata(tgroup)tgroup=6   -2.408  0.01606 *  
## ConditionG1:strata(tgroup)tgroup=6   0.638  0.52331    
## ConditionG2:strata(tgroup)tgroup=6      NA       NA    
## ---
## Signif. codes:  0 '***' 0.001 '**' 0.01 '*' 0.05 '.' 0.1 ' ' 1
## 
##                                    exp(coef) exp(-coef) lower .95 upper .95
## TrialNo                               1.0066     0.9934   1.00528    1.0080
## DistFromCentre                        0.9995     1.0005   0.99945    0.9996
## ConditionS:strata(tgroup)tgroup=1     0.3334     2.9996   0.08339    1.3328
## ConditionG1:strata(tgroup)tgroup=1    0.1666     6.0016   0.02005    1.3845
## ConditionG2:strata(tgroup)tgroup=1        NA         NA        NA        NA
## ConditionS:strata(tgroup)tgroup=2     1.4644     0.6829   1.28785    1.6652
## ConditionG1:strata(tgroup)tgroup=2    1.0960     0.9124   0.94082    1.2768
## ConditionG2:strata(tgroup)tgroup=2        NA         NA        NA        NA
## ConditionS:strata(tgroup)tgroup=3     1.3214     0.7568   1.15311    1.5141
## ConditionG1:strata(tgroup)tgroup=3    1.0151     0.9851   0.87300    1.1803
## ConditionG2:strata(tgroup)tgroup=3        NA         NA        NA        NA
## ConditionS:strata(tgroup)tgroup=4     1.2246     0.8166   1.14950    1.3045
## ConditionG1:strata(tgroup)tgroup=4    1.0562     0.9468   0.98691    1.1304
## ConditionG2:strata(tgroup)tgroup=4        NA         NA        NA        NA
## ConditionS:strata(tgroup)tgroup=5     1.0230     0.9775   0.98861    1.0587
## ConditionG1:strata(tgroup)tgroup=5    0.9544     1.0477   0.92420    0.9857
## ConditionG2:strata(tgroup)tgroup=5        NA         NA        NA        NA
## ConditionS:strata(tgroup)tgroup=6     0.8710     1.1481   0.77844    0.9747
## ConditionG1:strata(tgroup)tgroup=6    1.0346     0.9665   0.93190    1.1487
## ConditionG2:strata(tgroup)tgroup=6        NA         NA        NA        NA
## 
## Concordance= 0.553  (se = 0.002 )
## Likelihood ratio test= 516.3  on 14 df,   p=<2e-16
## Wald test            = 486.1  on 14 df,   p=<2e-16
## Score (logrank) test = 519.2  on 14 df,   p=<2e-16,   Robust = 306.9  p=<2e-16
## 
##   (Note: the likelihood ratio and score tests assume independence of
##      observations within a cluster, the Wald and robust score tests do not).
```

```
#ggcoxdiagnostics(tdmodel1_unique, type="schoenfeld") #straighter line for diagnostics - and better than other time splits

#As for original analysis, stratify the data by distance from centre instead.
#prepare data by splitting distance from centre into quartiles
summary(survival_data_repeat_exp2_3$DistFromCentre)#new values for changed datatset
```

```
##    Min. 1st Qu.  Median    Mean 3rd Qu.    Max. 
##   4.924 345.960 491.296 498.115 665.664 957.563
```

```
survival_data_repeat_exp2_3$DistFromCentreQuart<-rep(0,length(survival_data_repeat_exp2_3$Subject))
for (i in 1:length(survival_data_repeat_exp2_3$Subject)){
  if(survival_data_repeat_exp2_3$DistFromCentre[i]<=345.960){
    survival_data_repeat_exp2_3$DistFromCentreQuart[i]<-1
  }
  else if(survival_data_repeat_exp2_3$DistFromCentre[i]>345.960 && survival_data_repeat_exp2_3$DistFromCentre[i]<=491.296){
    survival_data_repeat_exp2_3$DistFromCentreQuart[i]<-2
  }
  else if(survival_data_repeat_exp2_3$DistFromCentre[i]>491.296 && survival_data_repeat_exp2_3$DistFromCentre[i]<=665.664){
    survival_data_repeat_exp2_3$DistFromCentreQuart[i]<-3
  }
  else{survival_data_repeat_exp2_3$DistFromCentreQuart[i]<-4}
}

# best split, following plot, and with low deviations for prop hazards plots (though cox.zph p values still break assumption)
SURVDATA2_MOBEX_SPLIT_unique<-survSplit(Surv(Time, Hit) ~ Condition+TrialNo+ParticipantNum+DistFromCentreQuart, data=survival_data_repeat_exp2_3, cut=c(0.6, 1.2,1.4,2,10.5), episode="tgroup", id="id")

tdmodel2_unique<-coxph(Surv(tstart,Time,Hit)~Condition:strata(tgroup)+TrialNo+strata(DistFromCentreQuart), cluster = ParticipantNum,data=SURVDATA2_MOBEX_SPLIT_unique) 

ggcoxdiagnostics(tdmodel2_unique, type="schoenfeld")
```

```
#Next, we test the significance of each fixed effect, and relevel the model by condition to extract hazard ratios.  

# test effects of trial number and condition:time - effects as for full dataset
tdmodel2b_unique<-coxph(Surv(tstart,Time,Hit)~Condition:strata(tgroup)+strata(DistFromCentreQuart), cluster = ParticipantNum,data=SURVDATA2_MOBEX_SPLIT_unique)
tdmodel2c_unique<-coxph(Surv(tstart,Time,Hit)~TrialNo+strata(DistFromCentreQuart), cluster =  ParticipantNum,data=SURVDATA2_MOBEX_SPLIT_unique)
```

Both trial number and time-dependent condition have a significant effect on survival:

```
# full model
AIC(tdmodel2_unique)
```

```
## [1] 456597.3
```

```
# effect of trial
AIC(tdmodel2b_unique)
```

```
## [1] 456729.6
```

```
# effect of condition
AIC(tdmodel2c_unique)
```

```
## [1] 456691.6
```

## EXPERIMENT 1B

This experiment is a classroom-based version of Experiment 1, with some small modifications (see Supplementary material).

### Load and prepare data

The data contains plays from 3 separate sessions.

```
data1B<-read.csv("Results_Expt1B.csv", header=TRUE)

# Find out how many players took part
length(unique(data1B$Date))#identify participants through time stamp

# Select only the last row for survival analyses (final click)
survival_data1B <- data1B %>%
  group_by(Date,TrialNo) %>%
  slice(n()) %>%
  ungroup()

# Replace NAs
survival_data1B$Time <- survival_data1B$Time %>% replace_na(30000)
survival_data1B$Time <- (survival_data1B$Time)/1000

# Make condition a factor
survival_data1B$fCondition <- factor(survival_data1B$Condition)
survival_data1B$fBackgroundType <- factor(survival_data1B$BackgroundType)

# Collapse across categories - to group all full specialists, part-generalists and generalists
survival_data1B <- survival_data1B %>%
  mutate(ConditionJoint = fct_collapse(fCondition, full_specialist = c("100", "0"),
                                       full_generalist = "50",
                                       part_specialist = c("25", "75")))

# Make condition a factor
survival_data1B$fConditionJoint <- factor(survival_data1B$ConditionJoint)
survival_data1B$TrialNo <- as.numeric(survival_data1B$TrialNo)
```

There were 63 participants in total.

### Odds ratios

Data for odds ratios:

```
survival_data_OR_Expt1B<- survival_data1B %>%
  group_by(fConditionJoint) %>%
  summarise(Total = n(), Total_hits = sum(Hit))
```

Targets and hits in Experiment 1B

| fConditionJoint | Total | Total\_hits |
| --- | --- | --- |
| full\_specialist | 756 | 677 |
| part\_specialist | 756 | 688 |
| full\_generalist | 378 | 355 |

Odds ratio calculations:

```
#Odds ratios relative to full specialists
condition <- c("full_specialist", "part_specialist", "generalist")
hit <- c("Yes", "No")
dat1b <- matrix(c(677, 79, 688, 68, 355, 23), nrow = 3, ncol = 2, byrow = TRUE)
dimnames(dat1b) <- list("Condition" = condition, "Hit" = hit)
or_expt1B<-oddsratio(dat1b)
```

Odds ratios relative to full specialists

|  | estimate | lower | upper |
| --- | --- | --- | --- |
| full\_specialist | 1.000 | NA | NA |
| part\_specialist | 0.847 | 0.601 | 1.192 |
| generalist | 0.558 | 0.337 | 0.890 |

Statistical tests for odds ratios (relative to full specialists)

|  | midp.exact | fisher.exact | chi.square |
| --- | --- | --- | --- |
| full\_specialist | NA | NA | NA |
| part\_specialist | 0.341 | 0.385 | 0.340 |
| generalist | 0.014 | 0.015 | 0.015 |

### Survival analyses

As in Experiment 1, an initial model is fitted with trial number and target condition as fixed effects, clustered by participant, but this breaks the proportional hazards assumption. A new model is fitted with condition as a time-dependent coefficient, improving the diagnostics.

```
# Initial model - assumptions not met
model_fit <- coxph(Surv(Time, Hit) ~ fConditionJoint + TrialNo + frailty(Date), data = survival_data1B)
```

```
cox.zph(model_fit) #assumption broken
```

```
##                   chisq    df       p
## fConditionJoint 23.0517  1.99 9.7e-06
## TrialNo          0.0565  1.00    0.81
## GLOBAL          23.0918 43.91    1.00
```

```
# Use survSplit to create time-dependent model
model_split <- survSplit(Surv(Time, Hit) ~ fConditionJoint + Date + TrialNo,
                         data = survival_data1B, cut = c(1.5,3.5,4.5), episode = "time_group")

fit_split <- coxph(Surv(tstart, Time, Hit) ~ fConditionJoint:strata(time_group) + TrialNo, cluster = Date, data = model_split)
```

```
cox.zph(fit_split) #assumption now respected
```

```
##                                     chisq df    p
## TrialNo                             0.115  1 0.73
## fConditionJoint:strata(time_group) 11.093  8 0.20
## GLOBAL                             11.178  9 0.26
```

```
# Null model
fit_split_null <- coxph(Surv(tstart, Time, Hit) ~ TrialNo, cluster = Date, data = model_split)

fit_split_null_2 <- coxph(Surv(tstart, Time, Hit) ~ fConditionJoint:strata(time_group), cluster = Date, data = model_split)


# Summary of full model
summary(fit_split)

# Relevel to extract all hazard ratios
model_split$fConditionJoint <- relevel(model_split$fCondition, "full_generalist")
fit_split <- coxph(Surv(tstart, Time, Hit) ~ fConditionJoint:strata(time_group) + TrialNo, cluster = Date, data = model_split)
summary(fit_split)

model_split$fConditionJoint <- relevel(model_split$fCondition, "part_specialist")
fit_split <- coxph(Surv(tstart, Time, Hit) ~ fConditionJoint:strata(time_group) + TrialNo, cluster = Date, data = model_split)
summary(fit_split)
```

Time-dependent condition has a significant effect on survival, but trial does not:

```
# full model
AIC(fit_split)
```

```
## [1] 23320.57
```

```
# effect of condition
AIC(fit_split_null)
```

```
## [1] 23334.97
```

```
# effect of trial
AIC(fit_split_null_2)
```

```
## [1] 23319.48
```

### Plot survival curves for Experiment 1B

```
fit_graph <- survfit(Surv(Time, Hit) ~ fConditionJoint, data = survival_data1B, cluster = Date, type = "kaplan-meier")

ggsurvplot(fit_graph, data=survival_data1B, conf.int = TRUE, pval = FALSE, break.time.by = 2,
           legend.title = "Condition:",legend.labs = c("Full specialist", "Part specialist", "Generalist"), 
           palette = c("#E69F00", "#009E73", "#56B4E9"), font.x = 16, font.y = 16, font.tickslab = 10, font.legend = 14,
           xlab = "Time (s)")
```

**Supplementary Figure 2** Survival probability for full specialists, part-specialists and generalist targets in Experiment 1B.

## EXPERIMENT 2B

This experiment is a lab-based version of Experiment 2, in controlled conditions (see Supplementary material).

### Load and prepare data

```
DATA2B<-read.csv("Results_Expt2B.csv", header=TRUE) #one player per folder (1-120)

# Determine number of players
number_subjects<-DATA2B%>%
  summarise(n_distinct(SubjNum))

# Set time out to 20s exactly
for (i in 1: length(DATA2B$SubjNum)){
  if(DATA2B$TimeTakenSecs[i]>20){
    DATA2B$TimeTakenSecs[i]<-20 #replace any value >20 with 20 exactly
  }
}

# Data inspection reveals some players missed many targets...
DATA2B$SubjNum<-as.factor(DATA2B$SubjNum)
misses <- DATA2B %>%
  filter(Hit == 0) %>%
  count(SubjNum)
#hist(misses$n) #2 big outliers, with over 20 misses
misses$SubjNum[which(misses$n>=18)]#remove anything with more than half misses - subjects 26 and 32

# Make dataset excluding players 26 and 32 -now, in total, n=37 players
DATA2B_EX<-DATA2B%>%
  filter(SubjNum!=26&SubjNum!=32)

# Add distance from centre of the screen (centre x=960, centre y=540)
for(i in 1: length(DATA2B_EX$SubjNum)){
  DATA2B_EX$DistFromCentre[i]<-sqrt((DATA2B_EX$xMoth[i]-960)^2+(DATA2B_EX$yMoth[i]-540)^2)
}
```

Due to the interruption of data collection for the COVD-19 pandemic and lockdown restrictions, only 39 took part in this experiment. In addition, 2 players missed over half the targets, so their data is excluded, leaving 37 players.

### Odds ratios

Data for odds ratios:

```
# Data for odds ratio
survival_data_OR_Expt2B <- DATA2B_EX %>%
  group_by(Condition) %>%
  summarise(total = n(), sum_hit = sum(Hit))
```

Targets and hits in Experiment 2B

| Condition | total | sum\_hit |
| --- | --- | --- |
| G1 | 444 | 389 |
| G2 | 444 | 417 |
| S | 444 | 409 |

Odds ratio calculations:

```
# Odds ratio relative to S
condition <- c("S", "G1", "G2")
hit <- c("Yes", "No")
datB <- matrix(c(409, 35, 389, 55, 417, 27), nrow = 3, ncol = 2, byrow = TRUE)
dimnames(datB) <- list("Condition" = condition, "Hit" = hit)
or_expt2B<-oddsratio(datB)

# Odds ratios relative to G2
condition <- c("G2", "G1", "S")
hit <- c("Yes", "No")
datB <- matrix(c(417, 27, 389, 55, 409, 35), nrow = 3, ncol = 2, byrow = TRUE)
dimnames(datB) <- list("Condition" = condition, "Hit" = hit)
or_expt2B_G<-oddsratio(datB)
```

Odds ratios relative to S

|  | estimate | lower | upper |
| --- | --- | --- | --- |
| S | 1.000 | NA | NA |
| G1 | 1.649 | 1.059 | 2.598 |
| G2 | 0.758 | 0.446 | 1.274 |

Statistical tests for odds ratios (relative to S)

|  | midp.exact | fisher.exact | chi.square |
| --- | --- | --- | --- |
| S | NA | NA | NA |
| G1 | 0.027 | 0.034 | 0.026 |
| G2 | 0.296 | 0.357 | 0.292 |

Odds ratios relative to G2

|  | estimate | lower | upper |
| --- | --- | --- | --- |
| G2 | 1.000 | NA | NA |
| G1 | 2.176 | 1.356 | 3.571 |
| S | 1.320 | 0.785 | 2.242 |

Statistical tests for odds ratios (relative to G2)

|  | midp.exact | fisher.exact | chi.square |
| --- | --- | --- | --- |
| G2 | NA | NA | NA |
| G1 | 0.001 | 0.002 | 0.001 |
| S | 0.296 | 0.357 | 0.292 |

### Survival analyses

#### Prepare data for survival analyses

The dataset already includes only the final click per trial, and time is already measured in seconds.

```
# add start column (0) for model syntax
DATA2B_EX$Start<-rep(0,length(DATA2B_EX$SubjNum))

# set subject as factor
DATA2B_EX$SubjNum<-as.factor(DATA2B_EX$SubjNum)
```

#### Plot survival curves for Experiment 2B

```
DATA2B_EX$Condition<-as.factor(DATA2B_EX$Condition)
DATA2B_EX$Condition<-relevel(DATA2B_EX$Condition, ref="S")
fit <- survfit(Surv(TimeTakenSecs, Hit) ~ Condition, data = DATA2B_EX, cluster = SubjNum, type = "kaplan-meier")

ggsurvplot(fit, data = DATA2B_EX, conf.int = TRUE, pval = FALSE, break.time.by = 1, 
           palette = c("#E69F00", "#56B4E9","#0072B2"),
           legend.title = "Condition:", 
           legend.labs=c("S","G1","G2"),
           font.x = 24, font.y = 24, 
           font.tickslab = 20, font.legend = 18, xlab = "Time (s)")
```

**Supplementary Figure 3** Survival probability for specialist (S) and generalist (G1,G2) targets in Experiment 2B.

#### Survival models

As for Experiment 2, we start with a simple coxph model including trial number, condition and distance from the target to the centre of the screen as fixed effects, clustered by participant number. This model breaks the proportional hazards assumption, so we fit a second model with condition as a time-dependent variable, improving the model diagnostics.

```
# Basic coxph model
phmodel2<-coxph(Surv(Start,TimeTakenSecs,Hit)~Condition+TrialNum+DistFromCentre, cluster = SubjNum, 
                data=DATA2B_EX) 
summary(phmodel2)
```

```
cox.zph(phmodel2) #proportional hazards assumption violated for condition and distance from centre
```

```
##                chisq df       p
## Condition       25.5  2 2.8e-06
## TrialNum         3.4  1   0.065
## DistFromCentre  79.7  1 < 2e-16
## GLOBAL         107.4  4 < 2e-16
```

```
# make distance from centre a categorical variable - split by quartiles
summary(DATA2B_EX$DistFromCentre)
DATA2B_EX$DFCQuartile<-rep(0, length(DATA2B_EX$SubjNum))
for (i in 1:length(DATA2B_EX$SubjNum)){
  if(DATA2B_EX$DistFromCentre[i]<=368.7){
    DATA2B_EX$DFCQuartile[i]<-1
  }
  else if(DATA2B_EX$DistFromCentre[i]>368.7 && DATA2B_EX$DistFromCentre[i]<=539.7){
    DATA2B_EX$DFCQuartile[i]<-2
  }
  else if(DATA2B_EX$DistFromCentre[i]>539.7 && DATA2B_EX$DistFromCentre[i]<=740.6){
    DATA2B_EX$DFCQuartile[i]<-3
  }
  else {DATA2B_EX$DFCQuartile[i]<-4}
}

# Change model to stratify by distance from centre
phmodel2<-coxph(Surv(Start,TimeTakenSecs,Hit)~Condition+TrialNum+strata(DFCQuartile), cluster = SubjNum, 
                data=DATA2B_EX) 
summary(phmodel2)
```

```
cox.zph(phmodel2) #proportional hazards assumption still violated for condition
```

```
##           chisq df       p
## Condition 26.74  2 1.6e-06
## TrialNum   1.69  1    0.19
## GLOBAL    29.28  3 2.0e-06
```

```
# Time-dependent model
  #based on inspection of survival plot, lines cross at approx 4.5 and 7s

# Split data
DATA2B_SPLIT<-survSplit(Surv(TimeTakenSecs, Hit) ~ Condition+TrialNum+SubjNum+DFCQuartile, data=DATA2B_EX, cut=c(4.5,7), episode="tgroup", id="id")
head(DATA2B_SPLIT)
str(DATA2B_SPLIT)#make sure trial and subjnum are in correct format

# Run time-dependent model
tdmodel2<-coxph(Surv(tstart,TimeTakenSecs,Hit)~Condition:strata(tgroup)+TrialNum+strata(DFCQuartile), cluster = SubjNum,data=DATA2B_SPLIT) 
summary(tdmodel2)
```

```
#ggcoxdiagnostics(tdmodel2) #straighter line for diagnostics 
cox.zph(tdmodel2)
```

```
##                          chisq df     p
## TrialNum                  2.32  1 0.128
## Condition:strata(tgroup) 11.52  6 0.074
## GLOBAL                   14.25  7 0.047
```

```
# Test the effect of trial number and condition:time
tdmodel2b<-coxph(Surv(tstart,TimeTakenSecs,Hit)~Condition:strata(tgroup)+strata(DFCQuartile), cluster = SubjNum,data=DATA2B_SPLIT) 
tdmodel2c<-coxph(Surv(tstart,TimeTakenSecs,Hit)~TrialNum+strata(DFCQuartile), cluster =SubjNum,data=DATA2B_SPLIT) 


# relevel to identify hazard ratios for all conditions and time splits
summary(tdmodel2)
DATA2B_SPLIT$Condition<-factor(DATA2B_SPLIT$Condition, levels=c("S","G2","G1"))
tdmodel2<-coxph(Surv(tstart,TimeTakenSecs,Hit)~Condition:strata(tgroup)+TrialNum+strata(DFCQuartile), cluster = SubjNum,data=DATA2B_SPLIT) 
summary(tdmodel2)
DATA2B_SPLIT$Condition<-factor(DATA2B_SPLIT$Condition, levels=c("S","G1","G2"))
tdmodel2<-coxph(Surv(tstart,TimeTakenSecs,Hit)~Condition:strata(tgroup)+TrialNum+strata(DFCQuartile), cluster = SubjNum,data=DATA2B_SPLIT) 
summary(tdmodel2)
```

Both trial number and time-dependent condition have a significant effect on survival:

```
# full model
AIC(tdmodel2)
```

```
## [1] 12165.06
```

```
#  effect of trialno
AIC(tdmodel2b)
```

```
## [1] 12176.48
```

```
# effect of condition
AIC(tdmodel2c)
```

```
## [1] 12187.53
```
